# Supplementary material for: ATPase activity of DFCP1 controls selective autophagy
Source: Nat Commun. 2023 Jul 8;14:4051. doi: 10.1038/s41467-023-39641-9 (PMC10329651; doi:10.1038/s41467-023-39641-9)
Supplement: Supplementary file 1 — Supplementary Figs. 1–10 and Tables 1 and 2 [file 41467_2023_39641_MOESM1_ESM.pdf]

Supplementary Fig. S1: DFCP1 is an ancient protein.

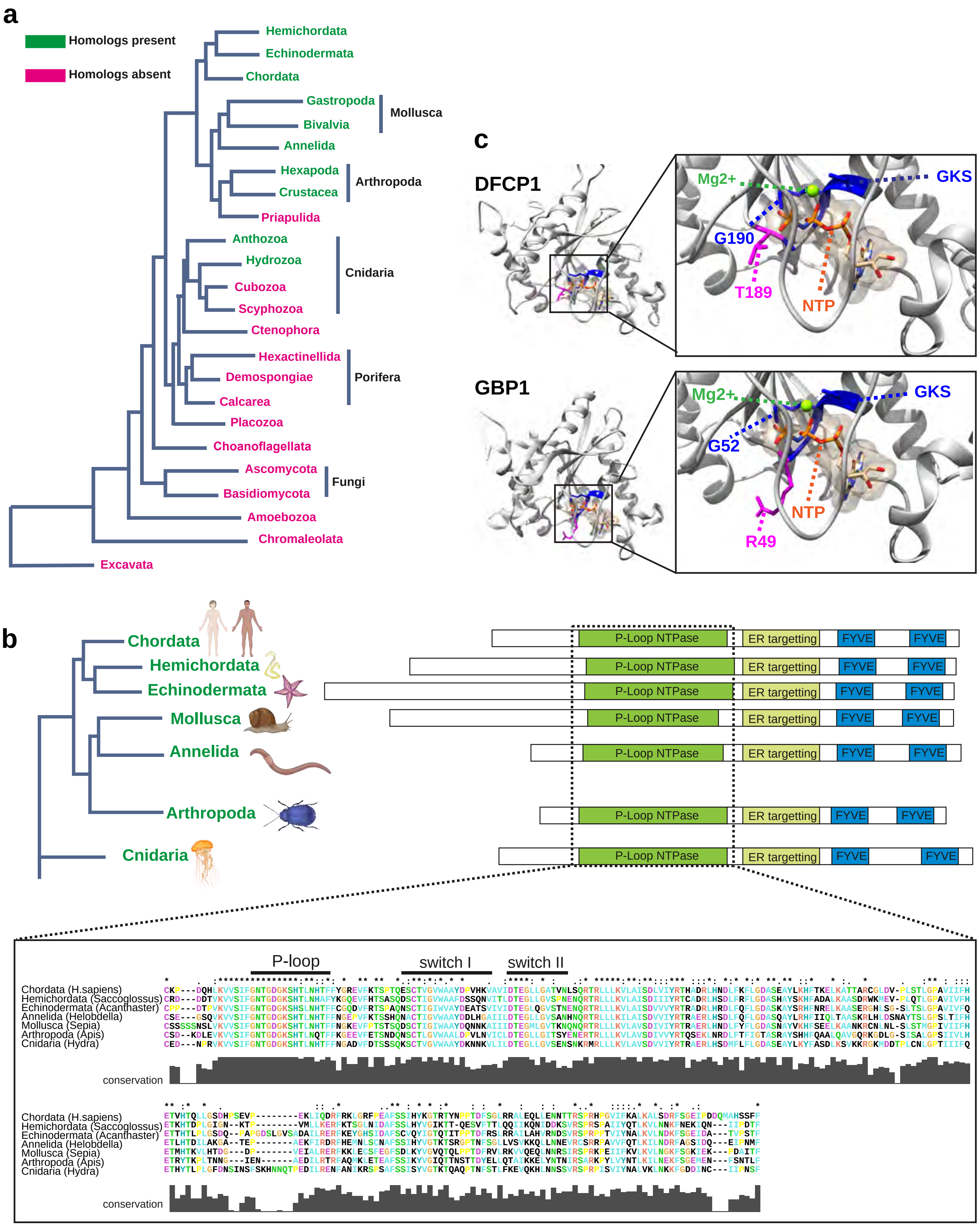

Supplementary Fig. S1: DFCP1 is an ancient protein.

(a) DFCP1 is conserved in the animal kingdom. Homologs of DFCP1 first occur in the cnidarian phylum and is present in nearly all metazoan phyla, indicating that it was first invented more than 0.5 billion years ago.

(b) The domain structure of DFCP1 is highly conserved throughout the metazoan tree of life. The DFCP1 domain architecture has remained largely unchanged from the first occurrence in the cnidarians. All homologs have a highly conserved ATP binding domain, an ER-binding domain and two FYVE domains. Created with biorender.com.

(c) Phyre2-generated homology model of DFCP1, based on the structure of GBP1. Candidates for amino acids required for nucleotide binding (GKS, residues 192-194) are indicated, and amino acids at the same position as the catalytic arginine of GBP1 (T189 in magenta, G190 in blue) and KRAS (G12, G13) are indicated.

Supplementary Fig. S2: DFCP1 binds and hydrolyses ATP and forms dimers.

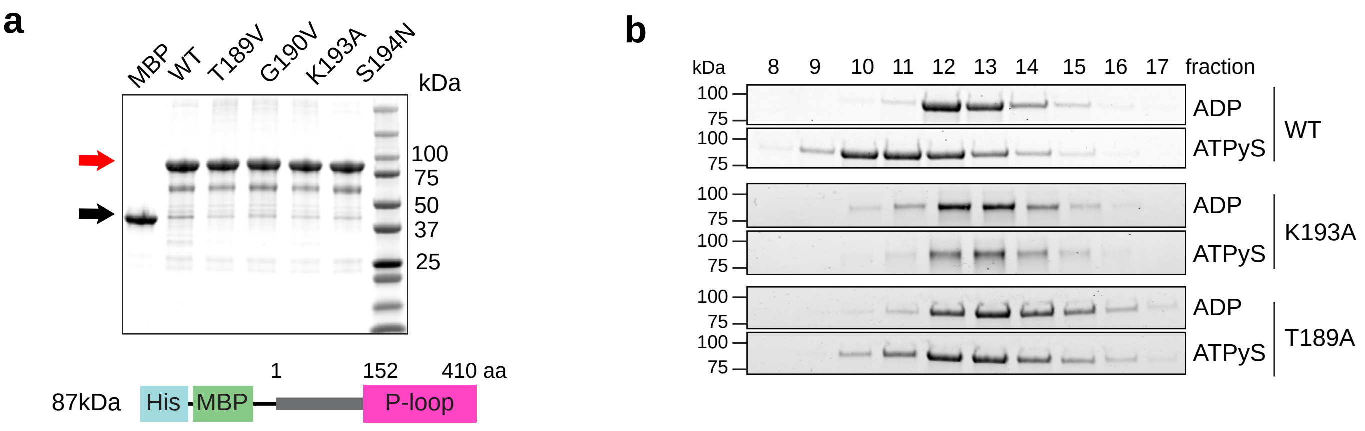

Supplementary Fig. S2: DFCP1 binds and hydrolyses ATP and forms dimers.

(a) Coomassie gel from purified DFCP1 N-terminus as used in Fig. 1c-k and Fig. 2b. Red arrow indicates MBP-DFCP1 for a calculated mass of 87 kDa, black arrow indicates MBP (43 kDa). Schematic overview of the His- and MBP-tagged N-terminus including P-loop of DFCP1 (DFCP1 K193A or DFCP1 T189A) used for nucleotide binding measurements.

(b) Coomassie gel showing individual 1 ml fractions of the size exclusion chromatography shown in Fig. 2b. One representative blot out of 3 independent experiments.

Supplementary Fig. S1: DFCP1 is an ancient protein.

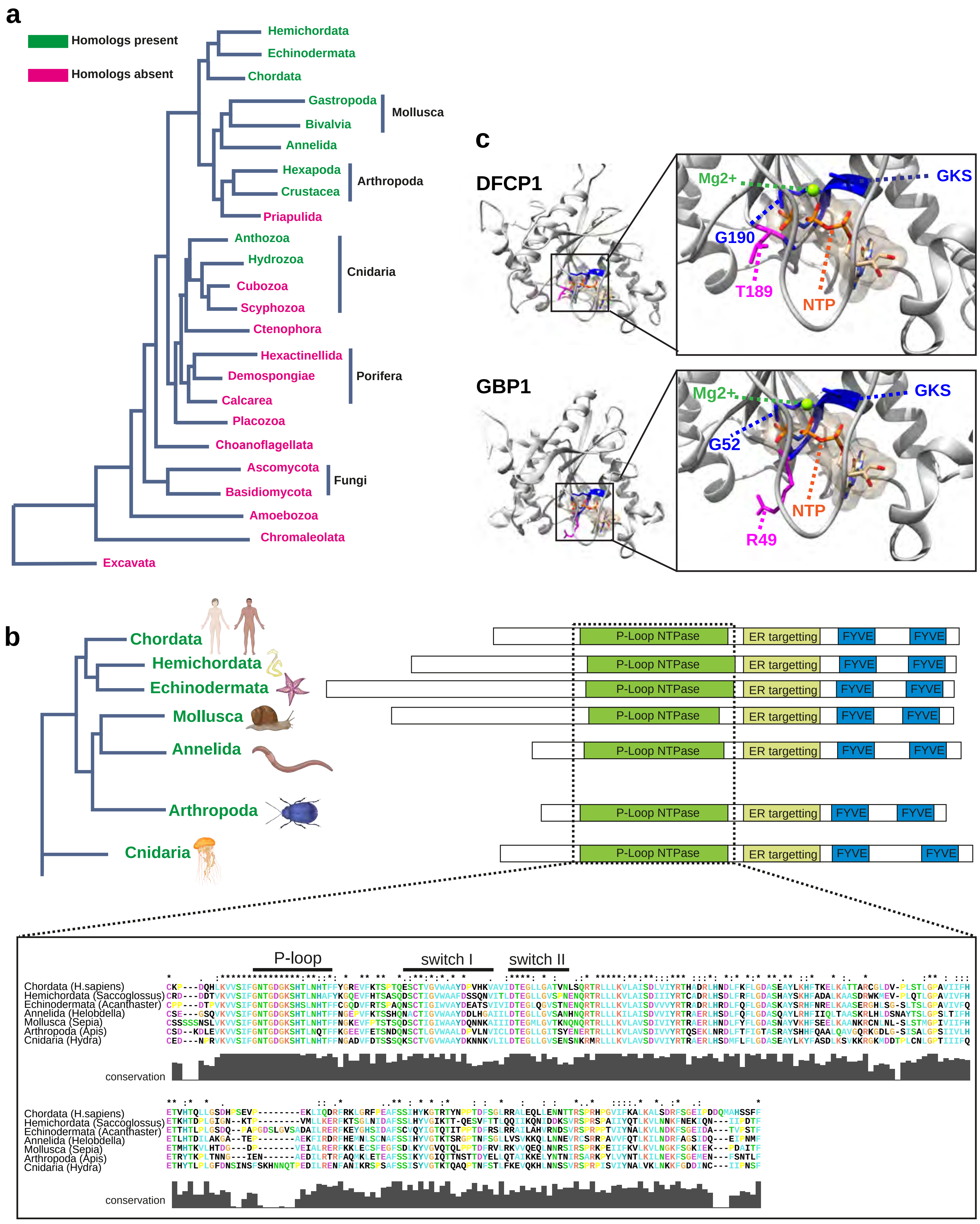

Supplementary Fig. S1: DFCP1 is an ancient protein.

(a) DFCP1 is conserved in the animal kingdom. Homologs of DFCP1 first occur in the cnidarian phylum and is present in nearly all metazoan phyla, indicating that it was first invented more than 0.5 billion years ago.

(b) The domain structure of DFCP1 is highly conserved throughout the metazoan tree of life. The DFCP1 domain architecture has remained largely unchanged from the first occurrence in the cnidarians. All homologs have a highly conserved ATP binding domain, an ER-binding domain and two FYVE domains.

(c) Phyre2-generated homology model of DFCP1, based on the structure of GBP1. Candidates for amino acids required for nucleotide binding (GKS, residues 192-194) are indicated, and amino acids at the same position as the catalytic arginine of GBP1 (T189 in magenta, G190 in blue) and KRAS (G12, G13) are indicated.

Supplementary Fig. S3: Characterization of DFCP1 KO- and kd- rescue systems.

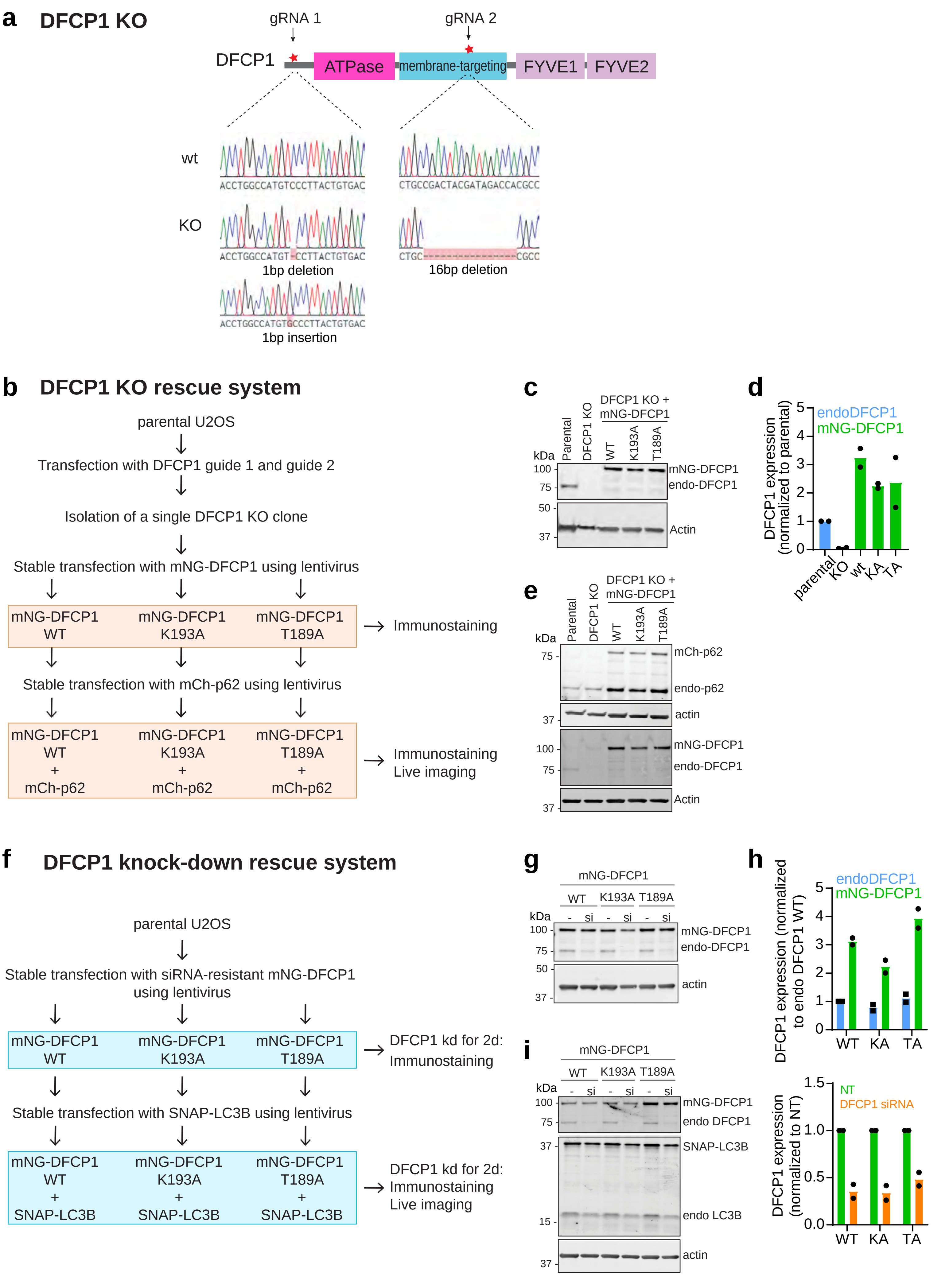

Supplementary Fig. S3: Characterization of DFCP1 KO- and knockdown-rescue systems.

(a) Generation and characterization of CRISPR/Cas9-mediated DFCP1 KO in U2OS cells. Shown are the localization of guide RNAs targeting DFCP1 and the sequences of a single clone which was isolated and validated by Sanger sequencing. This clone has been utilized for all further experiments.

(b) Schematic drawing of the generation of the DFCP1 KO rescue system. DFCP1 KO cells were stably transduced with a low titer of mNG-DFCP1 WT, K193A or T189A. These lines were further transduced with mCh-p62, and cells have been imaged live in EBSS for omegasome quantification (Fig. 3).

(c) Western blotting was used to measure protein levels of endogenous DFCP1 and mNG-DFCP1 using anti-DFCP1 antibody. Actin was used as loading control.

(d) Quantification of DFCP1 expression levels from (c), 2 independent experiments.

(e) Western blotting was used to measure protein levels of endogenous and exogenous expressed DFCP1 and p62 using anti-DFCP1 and anti-p62 antibodies. Actin was used as loading control. 2 independent experiments.

(f) Schematic drawing of the generation of the DFCP1 knockdown-rescue system for confocal imaging. U2OS cells were stably transduced with a low titer of siRNA-resistant mNG-DFCP1 WT, K193A or T189A. The resulting stable cell lines were then transfected with siRNA against DFCP1 for 2 d and treated as mentioned. Alternatively, cells were transduced with lentiviral vectors expressing SNAP-LC3B and cells were imaged live in EBSS for omegasome quantification (Supplementary Fig. S4).

(g) Western blotting was used to validate knockdown of DFCP1 using anti-DFCP1 antibody. Actin was used as loading control.

(h) Quantification of (g). Upper, knockdown-rescue lines express an approximately 3x level of endogenous DFCP1. Lower blot shows knockdown efficiency of DFCP1 siRNA. 2 independent experiments.

(i) Western blotting was used to measure protein levels of DFCP1 and LC3B, actin was used as loading control. One representative blot out of 2 independent experiments.

Supplementary Fig. S4: DFCP1 mutants are delayed in omegasome constriction.

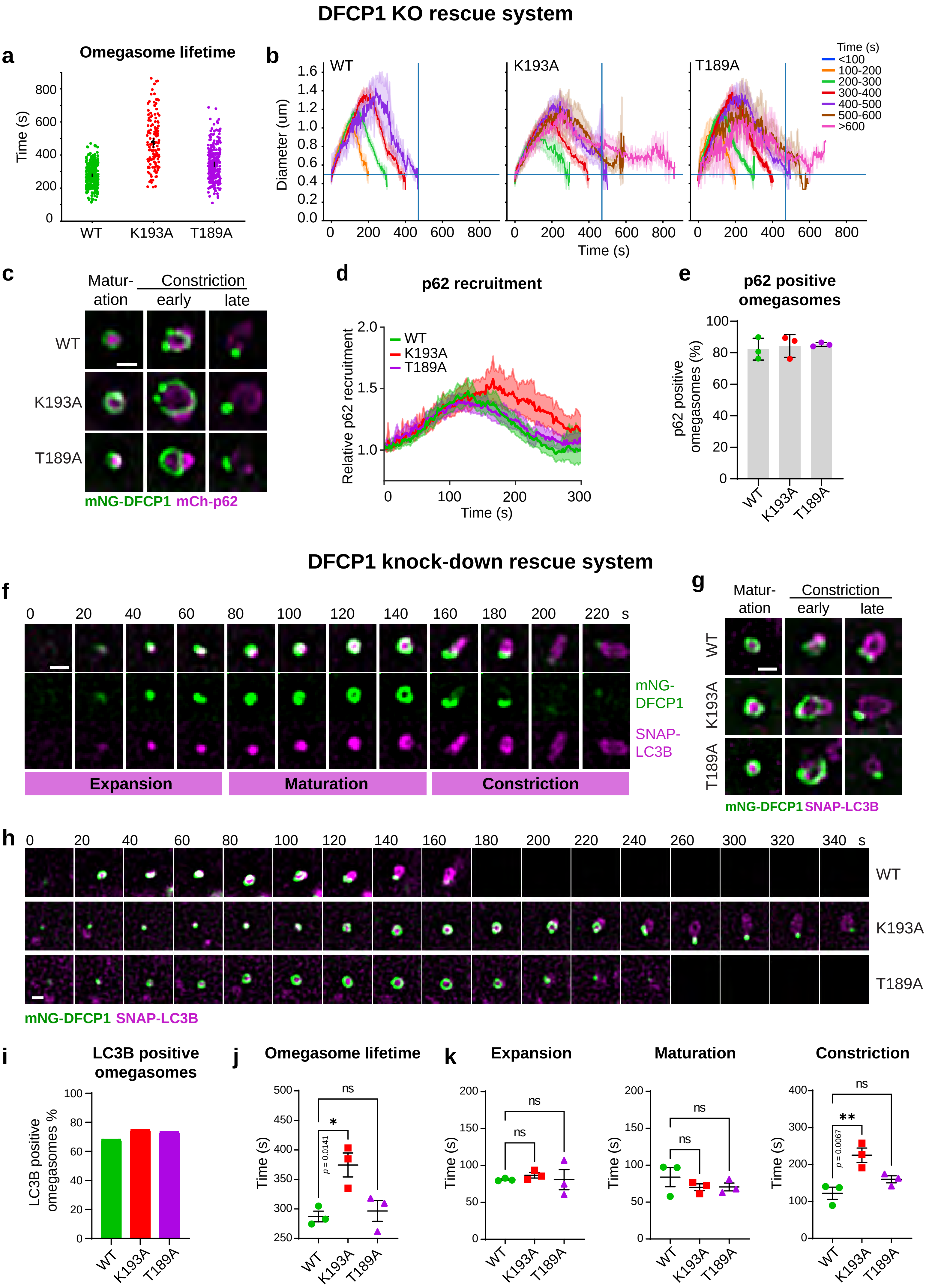

Supplementary Fig. S4: DFCP1 mutants are delayed in omegasome constriction.

U2OS DFCP1 KO lines stably expressing mNG-DFCP1 WT or mutant and mCh-p62. Cells were starved in EBSS and imaged live for 15 min every 2 s to capture omegasome formation. Same data set as Fig. 3.

(a) Lifetime of omegasomes in DFCP1 WT or mutant cells. Every dot represents an individual omegasome. Mean ± 95% CI. Same data set as Fig. 3f.

(b) Omegasomes have been grouped according to their lifetime. Plotted is the omegasome diameter over time. WT omegasomes typically disappear when constricted to a diameter of 0.5 µm (horizontal line). Vertical line indicates disappearance of all WT omegasomes. Mean ± 95% CI. Same data set as Fig. 3f.

(c) Examples of omegasomes. The first image shows the maturation phase, with p62 localizing in the middle. The second image shows the time point which was used as the onset of the constriction phase. A p62-positive autophagosome with a remaining DFCP1 dot is shown in the third image. Scale bar 0.5 µm. Representative image of > 20 events.

(d) The signal of DFCP1 was segmented, and mCh-p62 intensity was measured within a ROI around the center of the omegasome over time. 3 independent experiments. Mean ± 95% CI. Total number of tracks: WT (329), K193A (33), T189A (266).

(e) Same data set as in (d). Tracks where p62 intensity increased by at least 25% from the initial value within the first 200 s were scored as "p62 positive", tracks which failed to reach this threshold were designated as "negative". Bars: mean ± SD. 3 independent experiments. Total number of tracks: WT (329), K193A (133), T189A (266).

U2OS cells stably expressing siRNA resistant mNG-DFCP1 WT or mutant and SNAP-LC3B were depleted of endogenous DFCP1 by siRNA transfection for 2 d. Cells were starved in EBSS and imaged live for 15 min every 2 s to capture omegasome formation.

(f) Omegasome lifetime was divided into three phases as indicated. Shown are representative stills of omegasomes analyzed in (i-k). Scale bar 0.5 µm.

(g) Examples of omegasomes. The first image shows the maturation phase, with SNAP-LC3B localizing in the middle. The second image shows the time point which was defined as the onset of the constriction phase. A LC3B-positive autophagosome with a remaining DFCP1 dot is shown in the third image. Scale bar 0.5 µm. Shown are representative images of omegasomes analyzed in (i-k).

(h) Live imaging of total omegasome lifetime. Representative image of 3 independent experiments analyzed in (j). Scale bar 0.5 µm.

(i) Omegasomes have been blindly tracked, and if LC3B intensity was 1,5x over background, it has been noted as successful event. Total events: WT (44), K193A (53), T189A (56).

(j) Total time of an omegasome life cycle, with a DFCP1 spot as beginning and -end points. Each plotted point represents the mean value of one experiment. Mean ± SEM, 3 independent experiments. One-way ANOVA followed by Dunnett's multiple comparisons test, comparing against WT. Analyzed omegasomes: WT (93), K193A (45), T189A (89).

(k) Duration of individual phases of omegasome formation. Each plotted point represents the mean value of one experiment. The quantification is derived from the same raw data as (j). Mean ± SEM, 3 independent experiments. One-way ANOVA followed by Dunnett's multiple comparisons test,

Supplementary Fig. S5: Omegasome initiation is not affected in DFCP1 ATPase mutants.

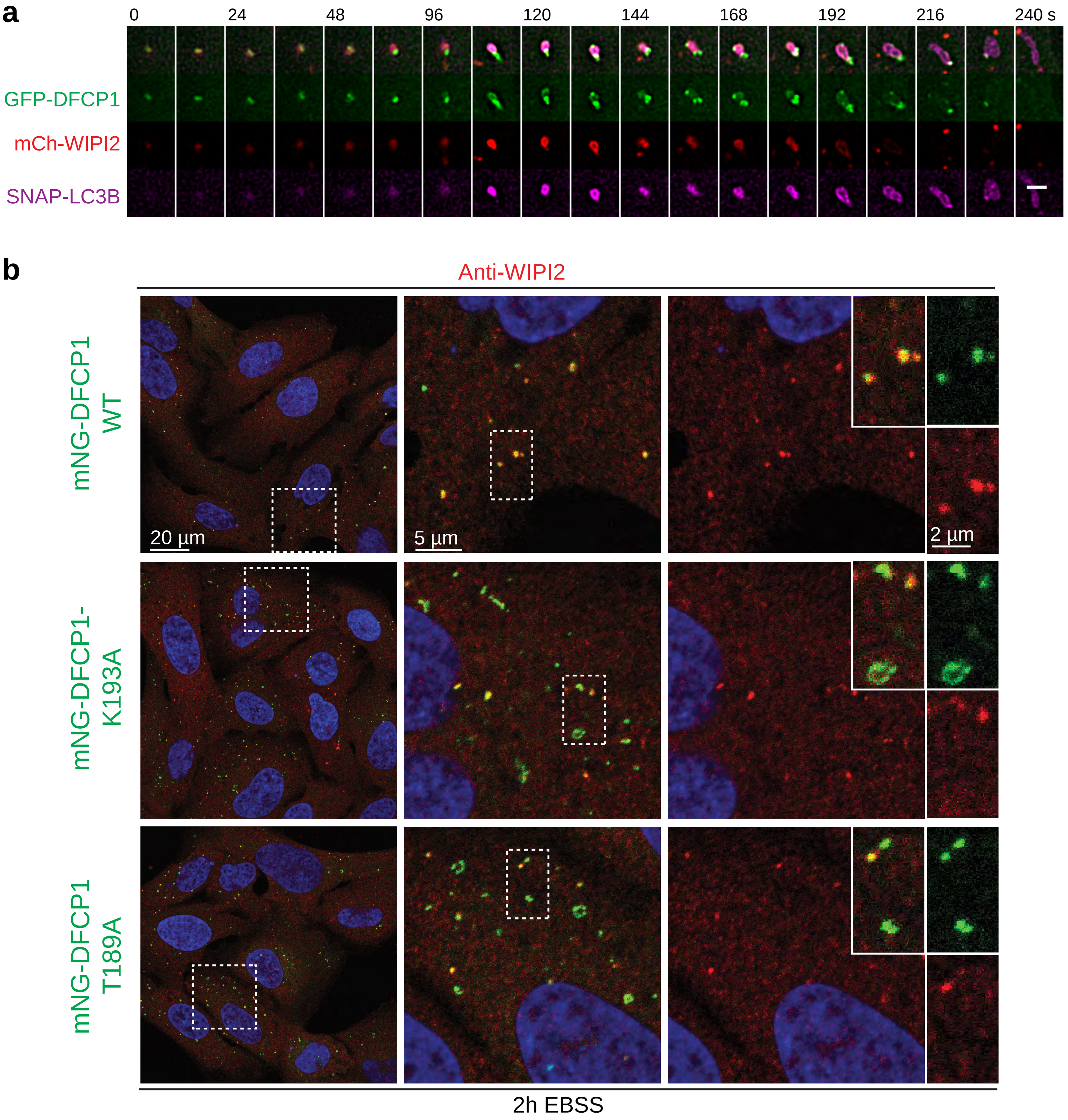

Supplementary Fig. S5: Omegasome initiation is not affected in DFCP1 ATPase mutants.

(a) U2OS cells expressing mNeongreen-DFCP1, mCherry-WIPI2 and SNAP-LC3B were incubated in EBSS for 15 min and imaged live to capture omegasome formation. Scale bar 1µm. Representative image of 53 events in total.

(b) Confocal images showing the localization and abundance of WIPI2 puncta in DFCP1 WT and mutant cells. U2OS cells stably expressing siRNA resistant mNG-DFCP1 WT, K193A or T189A, were depleted of endogenous DFCP1 by siRNA transfection for 2 d in complete medium before starvation for 2 h in EBSS. The cells were fixed, stained with anti-WIPI2 antibody, and analyzed by confocal microscopy. Insets show that some of the DFCP1-positive omegasomes co-localize with WIPI2. Scale bar 20 µm, insets 5 µm and 2 µm.

Supplementary Fig. S6: The majority of p62 puncta localizes with DFCP1-positive omegasomes.

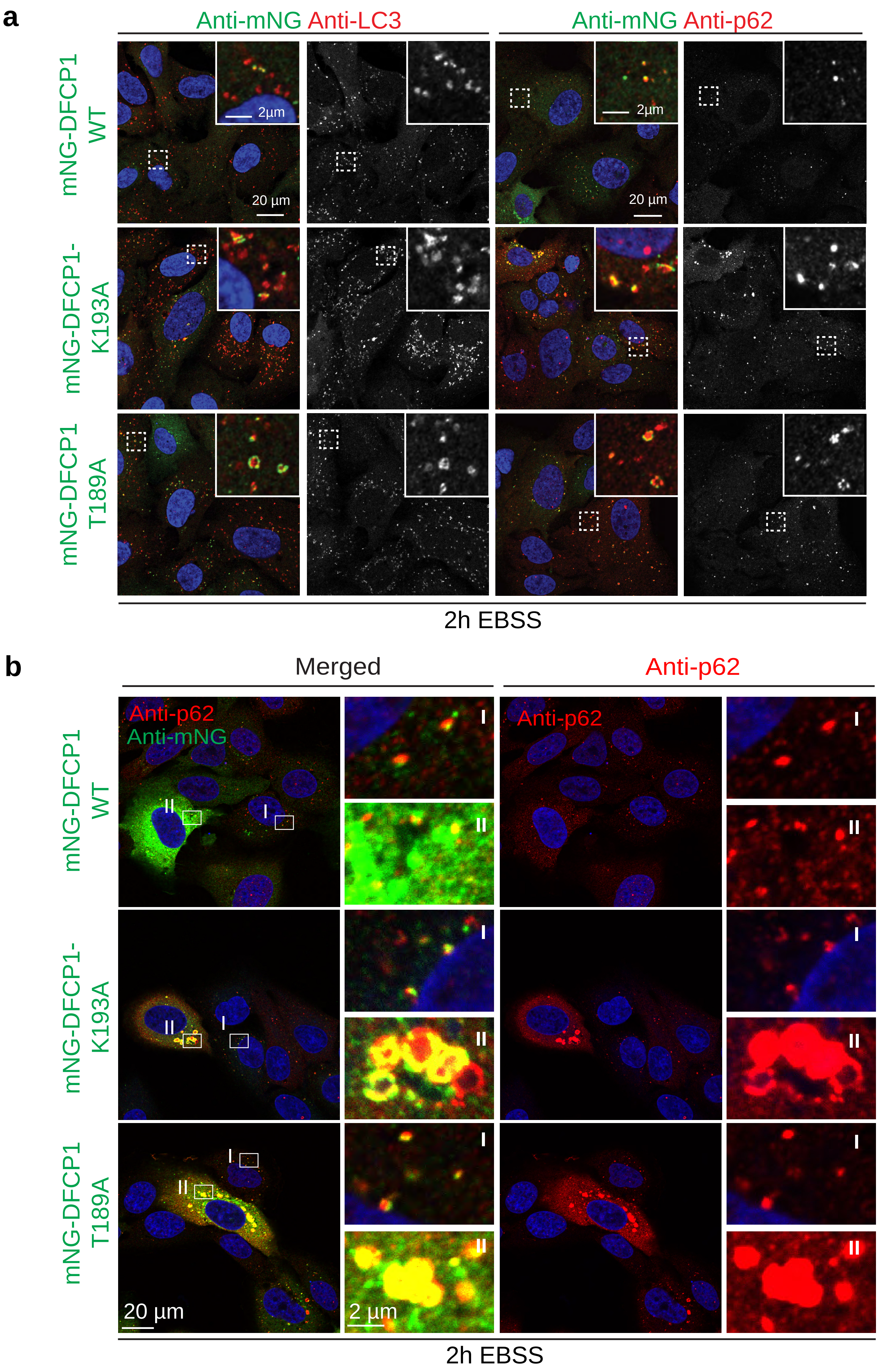

Supplementary Fig. S6: The majority of p62 puncta localizes with DFCP1-positive omegasomes

(a) Confocal images showing the localization of DFCP1, DFCP1 mutants, LC3B and p62 in DFCP1 KD rescue cell lines. U2OS cells stably expressing siRNA resistant mNG-DFCP1-WT, K193A, or T189A were depleted of endogenous DFCP1 by siRNA transfection for 2 d in complete medium before starvation for 2 h in EBSS. Cells were fixed, stained with anti-mNG and either anti-LC3B or anti-p62 antibodies and analyzed by confocal microscopy. Both LC3B and p62 are found in close association with DFCP1 positive omegasomes. Note that the localization of p62 is more restricted to omegasomes than LC3B. Quantifications in Fig. 4g, h. Scale bar 20 µm, inset 2 µm.

(b) U2OS cells stably expressing DFCP1 WT, K193A, T189A were depleted of endogenous DFCP1 by siRNA, treated with EBSS for 2 h, fixed and stained with anti-mNG and anti-p62 antibodies and analyzed by confocal microscopy. p62 colocalizes with DFCP1 in high and low expressing DFCP1 cells under all conditions. Notably, strong overexpression of the ATPase defective DFCP1 T189A allele causes hyper-accumulation of p62. Such cells, which appeared with low frequency in the stable cell lines, were not included in the high content-based image quantifications. Representative of > 5 images per condition.

Supplementary Fig. S7: In fed conditions, the levels of p62 are elevated in DFCP1-depleted cells.

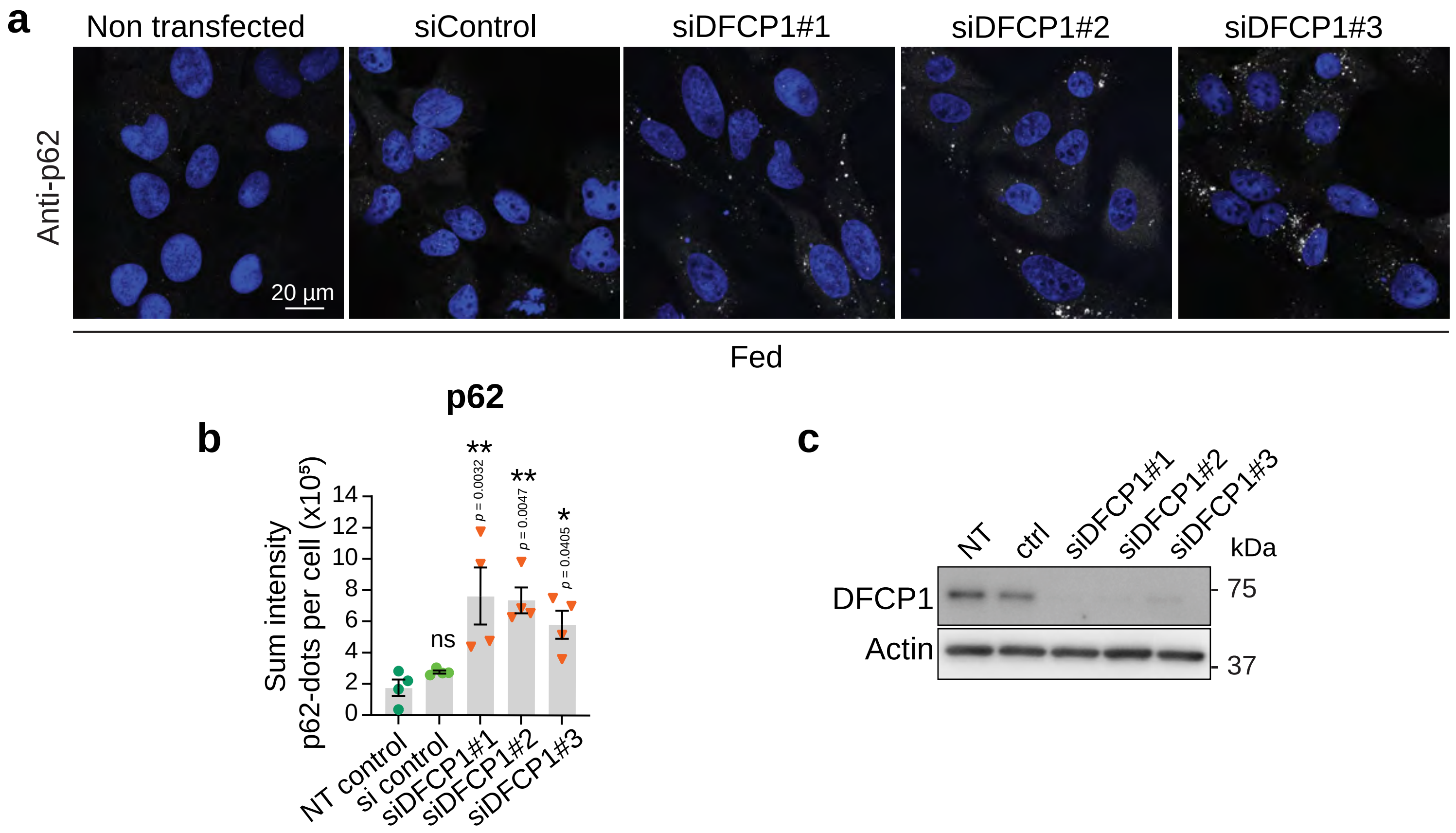

Supplementary Fig. S7: In fed conditions, the levels of p62 are elevated in DFCP1-depleted cells

(a) U2OS cells were depleted of endogenous DFCP1 by siRNA transfection for 2 d in complete medium, fixed, stained with anti-p62 antibody and Hoechst33342, and analyzed by confocal microscopy. Scale bar 20 µm.

(b) Quantifications of (a). Graphs represent quantification of the sum intensity of p62 dots per cell. Error bars denote mean ± SEM, 4 independent experiments. Each plotted point represents the mean value of one experiment. Ordinary one-way ANOVA, Dunnett's post hoc test; ns: not significant. Total cells analyzed per condition: NT (non-transfected): 575, siCtr (507), si#1 (464), si#2 (504), si#3 (507).

(c) Western blotting was used to validate knockdown of DFCP1 in (a, b) using anti-DFCP1 antibody. Actin was used as loading control. Representative blot from 4 independent experiments. Source data are provided as a Source data file.

Supplementary Fig. S8: Bulk autophagy is not affected in DFCP1 ATPase mutants.

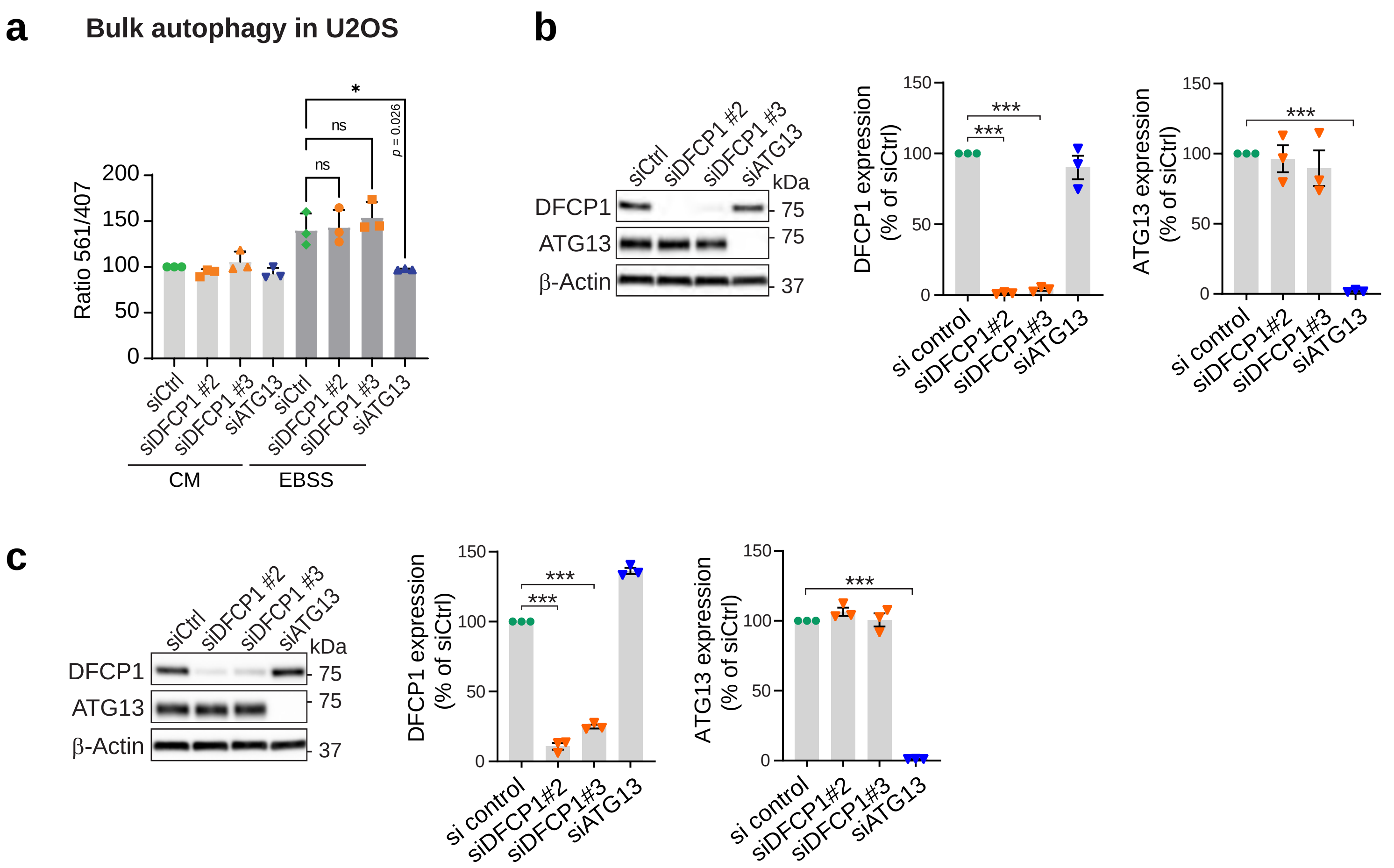

Supplementary Fig. S8: Bulk autophagy is not affected in DFCP1 ATPase mutants

(a) U2OS cells induced to express free cytoplasmic mKeima were transfected with control-, DFCP1- or ATG13 siRNA for 2 d. Cells were either grown for 16 h in complete medium (CM) or starved in EBSS. Cells were harvested and subjected to Flow Cytometry to determine the ratio of lysosome-localized mKeima to free, cytoplasmic mKeima. Values were normalized to siCtrl CM. Bars: ± SEM, 3 independent experiments. Statistics: One-way ANOVA followed by Dunnett's multiple comparisons test; p = 0.026 (EBSS siATG13).

(b) Knockdown validation for (a) with Western Blot and quantification of DFCP1- and ATG13 bands using Actin as a loading control. Values were normalized to control siRNA. Bars: ± SEM, 3 independent experiments. Statistics: Welch's test, comparing against siCtrl.

(c) Knockdown validation for Fig. 5a (bulk autophagy, LDHB-Keima in RPE) with Western Blot and quantification of DFCP1- and ATG13 bands using Actin as a loading control. Values were normalized to control siRNA. Bars: ± SEM, 3 independent experiments. Statistics: One sample t-test, comparing against a theoretical mean of 100.

Supplementary Fig. S9: DFCP1 ATPase regulates selective types of autophagy.

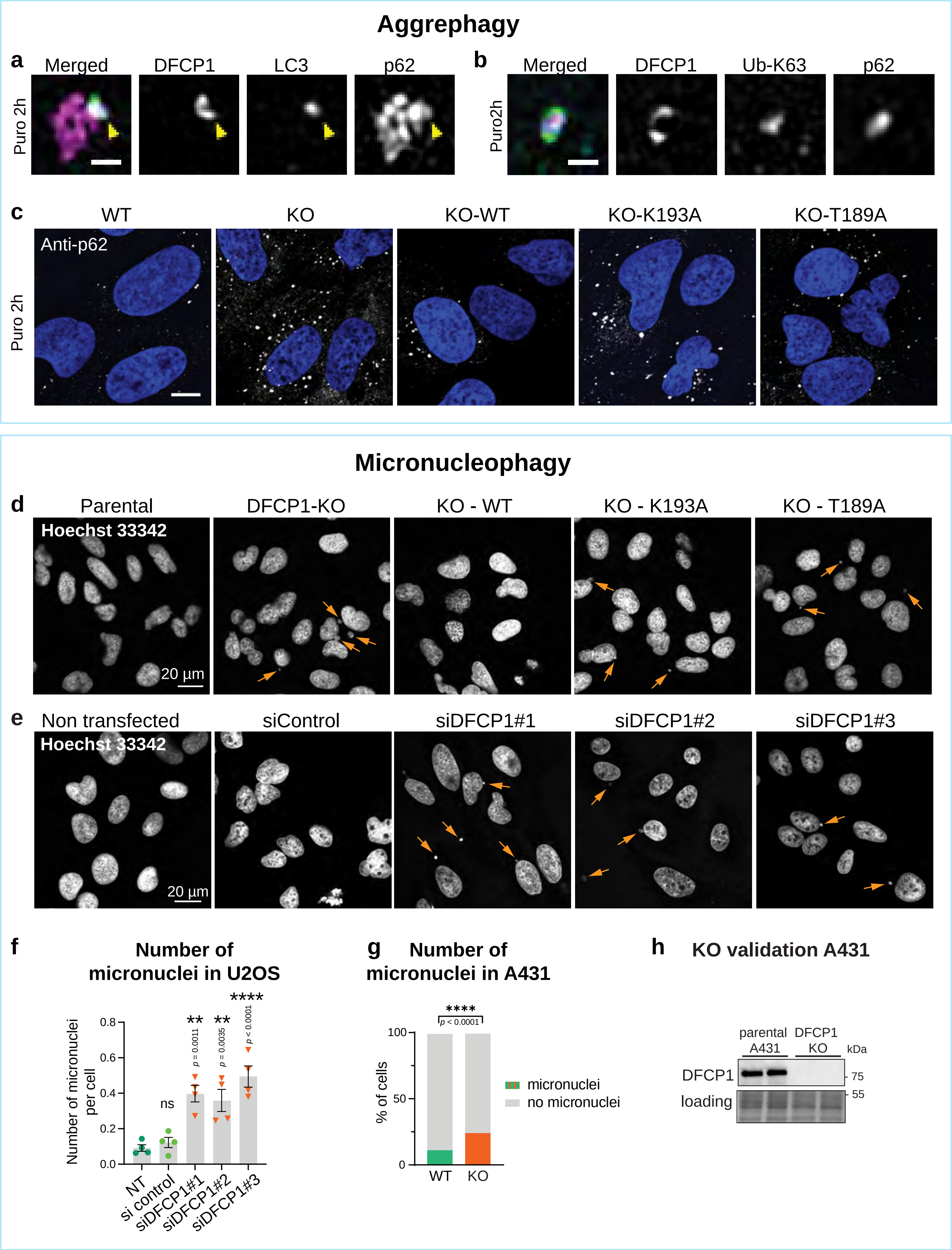

Supplementary Fig. S9: DFCP1 ATPase regulates selective types of autophagy.

(a) U2OS stably expressing mNG-DFCP1 WT were treated with 2.5 µg/ml Puromycin for 2 h, fixed, stained with anti-p62 and anti-LC3B antibodies, and analyzed by super-resolution microscopy. Arrowheads denote omegasome rings with a lumen. Scale bar: 0.5 µm. Representative image of 17 events.

(b) Cells treated as in (a) were stained with anti-p62 and anti-ubiquitin K63 antibodies and analyzed by super-resolution microscopy. Representative image of 9 events. Scale bar: 0.5 µm.

(c) Parental U2OS, DFCP1 KO and KO rescue lines stably expressing mNG-DFCP1 WT, K193A or T189A were treated with 2.5 µg/ml Puromycin for 2 h, fixed, stained with anti-p62 antibody, and analyzed by confocal microscopy. Quantifications in Fig. 5d. Scale bar: 10 µm.

(d) U2OS parental cells, DFCP1-KO and KO rescue lines stably expressing mNG-DFCP1 WT, K193A or T189A were grown in complete medium, fixed, stained with Hoechst 33342, and analyzed by confocal microscopy. Arrows point to micronuclei. Quantifications in Fig. 5i. Scale bar: 20 µm. Same images as in Fig. 4k, which show p62 accumulation upon DFCP1 KO.

(e) Parental U2OS cells were depleted of endogenous DFCP1 by siRNA transfection for 2 d in complete medium, fixed, stained with Hoechst33342, and analyzed by confocal microscopy. Arrows point to micronuclei. Same images as in Supplementary Fig. S7a, which show p62 accumulation upon DFCP1 depletion.

(f) Quantification of number of micronuclei per cell from (e). Error bars denote mean ± SEM, 4 independent experiments. Each plotted point represents the mean value of one experiment. One-way ANOVA, Dunnett's post hoc test; ns: not significant. Cells analyzed per condition: NT (575), siCtr (507), si#1 (464), si#2 (504), si#3 (507). Re-analysis of the images from dataset Supplementary Fig. S7a for scoring of micronuclei.

(g) Quantification of number of micronuclei in parental A431 and DFCP1 KO cells. Cells have been grown in complete medium, fixed and stained with DAPI, and analysed by widefield microscopy. Two-sided Fisher's exact test. Data pooled from 2 independent experiments. Cells analysed in total: WT (1230), KO (1148).

(h) DFCP1 KO validation using DFCP1 antibody. Parental A431 and KO clones have been loaded in duplicates. One representative blot out of 2 independent experiments.
Source data are provided as a Source data file.

Supplementary Fig. S10: DFCP1 ATPase regulates selective types of autophagy.

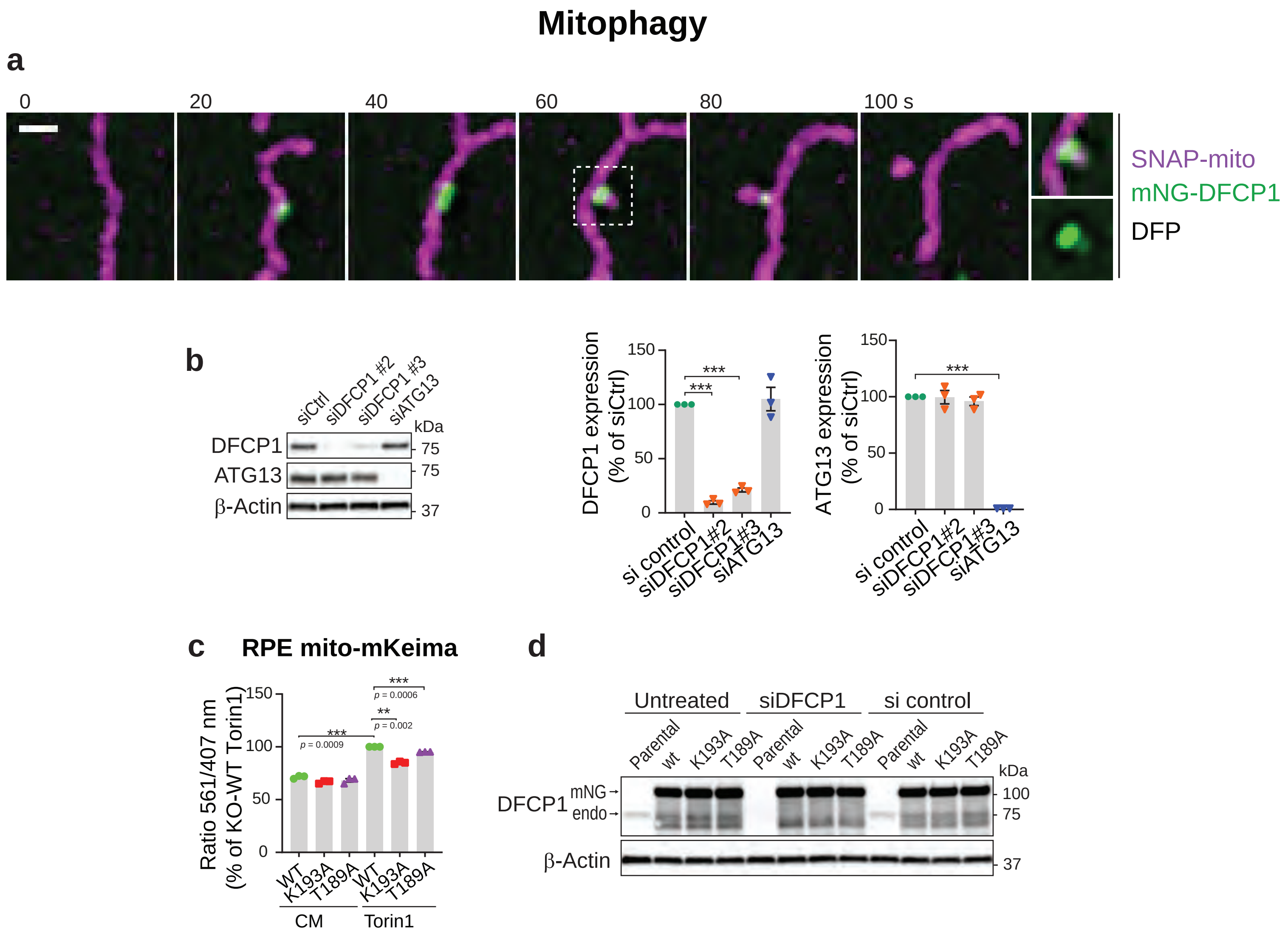

Supplementary Fig. S10: DFCP1 ATPase regulates selective types of autophagy.

(a) Example of a mitophagy event. U2OS cells stably expressing mNG-DFCP1 WT and SNAP-mito were treated for 18 h with 0.5 mM DFP and imaged live. Note the lumen of the omegasome ring, through which a part of the mitochondria is threaded. Scale bar: 1 µm. Representative movie for > 10 events.

(b) Knockdown validation for Fig. 5f (mitoKeima in RPE-1) with Western Blot. Quantification of DFCP1- and ATG13 bands using Actin as a loading control. Values were normalized to control siRNA. Bars: ±SEM, 3 independent experiments. Statistics: One sample t-test, comparing against a theoretical mean of 100.

(c) RPE-1 cells stably expressing mitochondrial mKeima probe and siRNA resistant mNG-DFCP1-WT, K193A, or T189A were depleted of endogenous DFCP1 by siRNA transfection for 2 d in complete medium (CM) before treatment with 50 nM Torin1 to induce mitophagy. Cells were harvested and analyzed by Flow Cytometry to determine the ratio of lysosome-localized mito-mKeima to free, cytoplasmic mito-mKeima. Values were normalized to the parental control. Bars: ± SEM, 3 independent experiments. Statistics: One-sample t-test, two-sided, comparing against a theoretical mean of 100.

(d) Knockdown validation for (c) with Western Blot.
Source data are provided as a Source data file.

Supplementary Table 1

Antibodies

Primary antibodies						
**Target**	**Species**	**Source**	**Identifier**	**Clone name**	**Lot number**	**Dilution**
DFCP1	rabbit	Cell Signaling Technology	mAb #85156	Clone E9R6P	Lot 1	WB 1:500
mNeonGreen	mouse	Chromotek	32f6-100	Clone 32F6	Lot 90425021AB-01	IF 1:500
GFP	mouse	Roche	11814460001	Clones 7.1 and 13.1	Lot 47859600	WB 1:500
beta-actin	mouse	Sigma-Aldrich	A5316	Clone AC-74	Lot 096M4855V	WB 1:20 000
LC3B	rabbit	MBL	PM036		Lot034	IF 1:500
LC3B	rabbit	Cell Signaling Technology	2775s		Lot12, Lot13	WB 1:1000, IF 1:100
WIPI2	mouse	abcam	ab105459	Clone 2A2	Lot GR3225460-2	IF 1:200
p62	rabbit	MBL	PM045		Lot022	WB 1:10 000, IF 1:500
p62	guinea pig	Progen	GP62-C		Lot 703241-03	IF 1:250
Ubiquitin	mouse	EMD Millipore Corp.	04-263	Clone FK2	Lot 3299923	IF 1:400
Ubiquitin-K63	rabbit	Millipore	05-1308	Clone Apu3	Lot 2012973	IF 1:200
Tom20	mouse	BD	612278	Clone 612278	Lot 8004895	IF 1:100
Atg13	rabbit	Cell Signaling Technology	#13468	Clone E1Y9V		WB 1:1000
**Secondary antibodies**						
**Target**	**Species**	**Source**	**Identifier**	**Clone name**	**Lot number**	**Dilution**
Alexa488-anti-mouse	donkey	Jackson	715-545-151		Lot 148532	1:500
Alexa488-anti-rabbit	donkey	Jackson	711-545-152		Lot 146871	1:500

Alexa568-anti-mouse	donkey	Molecular Probes	A10037		Lot 2420698	1:500
Alexa568-anti-rabbit	donkey	Molecular Probes	A10042		Lot 1891789	1:500
Alexa647-anti-mouse	donkey	Jackson	715-605-150		Lot 151088	1:500
Alexa647-anti-rabbit	donkey	Jackson	711- 605 - 152		Lot 145376	1:500
Alexa647-anti-guinea pig	donkey	Jackson	706-605-148		Lot 101663	1:500
anti- rabbit IgG HRP	goat	Bio-Rad	1706515		Lot 64371828	WB 1:2000
Anti-rabbit HRP	goat	Jackson	111-035-144		Lot 157511	WB 1:5000
Anti-mouse HRP	goat	Jackson	115-035-003		Lot 158821	WB 1:5000
anti-rabbit IRDye 680RD	donkey	LI-COR Biosciences	926-68073			WB 1:5000
anti-mouse IRDye 680RD	donkey	LI-COR Biosciences	926-68072			WB 1:5000
anti-goat IRDye 800CW	donkey	LI-COR Biosciences	926-32214			WB 1:5000
anti-rabbit IRDye 800CW	donkey	LI-COR Biosciences	926-32213			WB 1:5000
Anti-mouse IRDye 800CW	donkey	LI-COR Biosciences	926-32212			WB 1:5000

Supplementary Table 2

Constructs

pET-His6-MBP-TEV-LIC	Addgene # 29708
pET_His6_MBP_DFCP1-N-term_TEV_Lic	this study
pET_His6_MBP_DFCP1_T189V_TEV_Lic	this study
pET_His6_MBP_DFCP1_G190V_TEV_Lic	this study
pET_His6_MBP_DFCP1_K193A_TEV_Lic	this study
pET_His6_MBP_DFCP1_S194N_TEV_Lic	this study

pET_His6_MBP_DFCP1_T189A_TEV_Lic	this study
pFastBacDual_GFP / His6-DFCP1	this study
pCDH-PGK_mNeonGreen-DFCP1_siDFCP1-siRNAresistant_IRES_Puro	this study
pCDH-PGK_mNeonGreen-DFCP1_K193A_siDFCP1-siRNAresistant_IRES_Puro	this study
pCDH-PGK_mNeonGreen_DFCP1_T189A_siDFCP1-siRNAresistant_IRES_Puro	this study
pCDH-PGK_mCherry-DFCP1_IRES_Blast	this study
pCDH-PGK_Halo-DFCP1_siDFCP1-siRNAresistant_IRES_Blast	this study
pCDH-PGK_Halo-DFCP1_T189A_siDFCP1-siRNAresistant_IRES_Blast	this study
pCDH-PGK_Halo-DFCP1_K193A_siDFCP1-siRNAresistant_IRES_Blast	this study
pSpCas9(BB)-2A-Puro (PX459) V2.0	Addgene # 62988
pX459_DFCP1_guide_1+2	this study
pCDH-PGK_SNAP_LC3B_IRES_Neo	Zhen [1], Y *et al*, 2020
pCDH-PGK-mCherry_p62_Neo	Agudo-Canalejo [2], J. *et al,* 2021
pCDH-PGK-mCherry_WIPI2_IRES_Blast	this study
pCDH-PGK_Mito-mTagBFP2-3xSNAPtag_IRES_Neo	Zhen [1], Y *et al*, 2020
pEGFP-C2-DFCP1	Axe [3], E. L. *et al.* 2008

Supplementary References

1 Zhen, Y. *et al.* ESCRT-mediated phagophore sealing during mitophagy. *Autophagy* **16**, 826-841, doi:10.1080/15548627.2019.1639301 (2020).

2 Agudo-Canalejo, J. *et al.* Wetting regulates autophagy of phase-separated compartments and the cytosol. *Nature* **591**, 142-146, doi:10.1038/s41586-020-2992-3 (2021).

3 Axe, E. L. *et al.* Autophagosome formation from membrane compartments enriched in phosphatidylinositol 3-phosphate and dynamically connected to the endoplasmic reticulum. *J Cell Biol* **182**, 685-701, doi:10.1083/jcb.200803137 (2008).
