# Supplementary material for: ATPase activity of DFCP1 controls selective autophagy
Source: Nat Commun. 2023 Jul 8;14:4051. doi: 10.1038/s41467-023-39641-9 (PMC10329651; doi:10.1038/s41467-023-39641-9)
Supplement: Supplementary file 3 — Legends for Supplementary Movies 1–6 [file 41467_2023_39641_MOESM3_ESM.pdf]

Description of Additional Supplementary Files

File name: Supplementary Movie 1
Description: U2OS DFCP1 KO lines stably expressing mNG-DFCP1 WT and mCh-p62. Cells were starved in EBSS and imaged live to capture omegasome formation. Omegasome formation was divided into three phases as indicated in Fig. 3b. Quantification in Fig. 3d-g. Frame 2 sec. Scale bar 1 µm.

File name: Supplementary Movie 2
Description: corresponding to Fig. 3c. U2OS DFCP1 KO lines stably expressing mNG-DFCP1 WT or -mutant and mCh-p62. Cells were starved with EBSS and imaged live to capture omegasome formation. Quantification in Fig. 3d-g. Frame 2 sec. Scale bar 1 µm.

File name: Supplementary Movie 3
Description: corresponding to Supplementary Fig. S4f. U2OS DFCP1 knockdown/rescue lines stably expressing mNG-DFCP1 WT and SNAP-LC3B were depleted by endogenous DFCP1 for 2 d, starved with EBSS and imaged live to capture omegasome formation. Omegasome formation was divided into three phases as indicated in Supplementary Fig. S4f. Quantifications in Supplementary Fig. S4 j, k. Frame 2 sec. Scale bar 1 µm.

File name: Supplementary Movie 4
Description: corresponding to Supplementary Fig. S4h. U2OS DFCP1 knockdown/rescue lines stably expressing mNG-DFCP1 WT or -mutant and SNAP-LC3B were depleted by endogenous DFCP1 for 2 d, starved with EBSS and imaged live to capture omegasome formation. Quantifications in Supplementary Fig. S4j, k. Frame 2 sec. Scale bar 1 µm.

File name: Supplementary Movie 5
Description: corresponding to Fig. 5e. Example of a mitophagy event. U2OS cells stably expressing mNG-DFCP1 WT and SNAP-mito were treated for 18 h with 0.5 mM DFP and imaged live. The movie shows a piecemeal of mitochondria, which is engulfed by the phagophore. Note that the phagophore is weakly positive for mNG-DFCP1. Frame 2 sec. Scale bar 1 µm.

File name: Supplementary Movie 6
Description: corresponding to Supplementary Fig. S10a. Example of a mitophagy event. U2OS cells stably expressing mNG-DFCP1 WT and SNAP-mito were treated for 18 h with 0.5 mM DFP and imaged live. Note the lumen of the omegasome ring, through which a part of the mitochondria is threaded. Frame 2 sec. Scale bar 1 µm.
