# Supplementary material for: ATPase activity of DFCP1 controls selective autophagy
Source: Nat Commun. 2023 Jul 8;14:4051. doi: 10.1038/s41467-023-39641-9 (PMC10329651; doi:10.1038/s41467-023-39641-9)
Supplement: Supplementary file 10 — Reporting Summary [file 41467_2023_39641_MOESM10_ESM.pdf]

nature portfolio

Corresponding author(s): Nähse
Schink
Stenmark

Last updated by author(s): 2023/19/06

Reporting Summary

Nature Portfolio wishes to improve the reproducibility of the work that we publish. This form provides structure for consistency and transparency in reporting. For further information on Nature Portfolio policies, see our Editorial Policies and the Editorial Policy Checklist.

Statistics

For all statistical analyses, confirm that the following items are present in the figure legend, table legend, main text, or Methods section.

n/a	Confirmed	
☐	☒	The exact sample size (*n*) for each experimental group/condition, given as a discrete number and unit of measurement
☐	☒	A statement on whether measurements were taken from distinct samples or whether the same sample was measured repeatedly
☐	☒	The statistical test(s) used AND whether they are one- or two-sided<br>*Only common tests should be described solely by name; describe more complex techniques in the Methods section.*
☒	☐	A description of all covariates tested
☐	☒	A description of any assumptions or corrections, such as tests of normality and adjustment for multiple comparisons
☐	☒	A full description of the statistical parameters including central tendency (e.g. means) or other basic estimates (e.g. regression coefficient) AND variation (e.g. standard deviation) or associated estimates of uncertainty (e.g. confidence intervals)
☐	☒	For null hypothesis testing, the test statistic (e.g. *F*, *t*, *r*) with confidence intervals, effect sizes, degrees of freedom and *P* value noted<br>*Give P values as exact values whenever suitable.*
☒	☐	For Bayesian analysis, information on the choice of priors and Markov chain Monte Carlo settings
☒	☐	For hierarchical and complex designs, identification of the appropriate level for tests and full reporting of outcomes
☒	☐	Estimates of effect sizes (e.g. Cohen's *d*, Pearson's *r*), indicating how they were calculated

Our web collection on statistics for biologists contains articles on many of the points above.

Software and code

Policy information about availability of computer code

Data collection	Image acquisition was performed using Softworx software 7.0 (GE Healthcare), Zen Software 2010 (Zeiss) and MicroManager 1.4. Flow cytometry data was collected using BD FacsDiva software 8.0.3 (BD Biosciences). Structural predictions were performed using AlphaFold
Data analysis	Deconvolution was performed using Softworx 7.0 software.<br>Imaging data was analyzed using FIJI/Imagej (1.53c) and postprocessed using Python scripts.<br>Flow cytometry analysis was performed using BD FacsDiva software 8.0.3 and FlowJo™ software v10.8 (BD Biosciences, San Jose, CA, USA).<br>Image visualization was performed in Imaris 9.6 (Bitplane).<br>Data analysis, statistics and plotting was performed using Graphpad Prism (8.4) .<br>Structural data was analyzed using UCSF ChimeraX v.1.5 and AlphaFold2.<br><br>GitHub web-link: https://github.com/koschink/Naehse_et_al

For manuscripts utilizing custom algorithms or software that are central to the research but not yet described in published literature, software must be made available to editors and reviewers. We strongly encourage code deposition in a community repository (e.g. GitHub). See the Nature Portfolio guidelines for submitting code & software for further information.

Data

Policy information about availability of data

All manuscripts must include a data availability statement. This statement should provide the following information, where applicable:

- Accession codes, unique identifiers, or web links for publicly available datasets
- A description of any restrictions on data availability
- For clinical datasets or third party data, please ensure that the statement adheres to our policy

All data shown and used to generate plots, as well as detailed statistical information, accompanies this manuscript in the source data file. Uncropped and unprocessed western blots and gels are shown in source data files. Underlying image data are available from the corresponding authors (V.N., K.O.S, H.S.) upon request. Source data are provided with this paper. A data availability statement is included in the text.

Publicly available entries used in this study are PDB:1f5n, PDB: 3q5e, PDB: 621p, PDB:4obe.

Research involving human participants, their data, or biological material

Policy information about studies with human participants or human data. See also policy information about sex, gender (identity/presentation), and sexual orientation and race, ethnicity and racism.

Reporting on sex and gender	not applicable
Reporting on race, ethnicity, or other socially relevant groupings	not applicable
Population characteristics	not applicable
Recruitment	not applicable
Ethics oversight	not applicable

Note that full information on the approval of the study protocol must also be provided in the manuscript.

Field-specific reporting

Please select the one below that is the best fit for your research. If you are not sure, read the appropriate sections before making your selection.

☒ Life sciences ☐ Behavioural & social sciences ☐ Ecological, evolutionary & environmental sciences

For a reference copy of the document with all sections, see nature.com/documents/nr-reporting-summary-flat.pdf

Life sciences study design

All studies must disclose on these points even when the disclosure is negative.

Sample size	No statistical methods were used to pre-determine the correct sample size. All statistical calculations were derived from at least 3 biological replicates. For microscopy-based assays, each biological repeat included sufficient cell numbers to ensure that the effects could be robustly measured and was repeated at least 3 times
Data exclusions	Collected data was only excluded for technical reasons:- if verification of knockdown efficiency showed insufficient knockdown (less than 70 %). - Live-cell imaging data was excluded if it showed strong photobleaching or the imaged cells showed phototoxicity effects; in these cases, samples were excluded prior to analysis.
Replication	Data was successfully replicated in independent experiments (at least three independent experiments) and was reproduced by independent investigators.
Randomization	This study used cultured cell lines which are homogenous and isogenic, therefore, no randomization was used prior to treatment.
Blinding	Investigators were not blinded. Key experiments were repeated by independent researchers. To reduce potential human bias, quantitative measurements were performed using automated image processing algorithms. High content images were collected using automated systems, thus excluding any bias. For live cell imaging, cells were selected before their behavior was known.

Reporting for specific materials, systems and methods

We require information from authors about some types of materials, experimental systems and methods used in many studies. Here, indicate whether each material, system or method listed is relevant to your study. If you are not sure if a list item applies to your research, read the appropriate section before selecting a response.

Materials & experimental systems

n/a	Involved in the study
☐	☒ Antibodies
☐	☒ Eukaryotic cell lines
☒	☐ Palaeontology and archaeology
☒	☐ Animals and other organisms
☒	☐ Clinical data
☒	☐ Dual use research of concern
☒	☐ Plants

Methods

n/a	Involved in the study
☒	☐ ChIP-seq
☐	☒ Flow cytometry
☒	☐ MRI-based neuroimaging

Antibodies

Antibodies used

DFCP1 rabbit Cell Signaling Technology mAb #85156 Clone E9R6P Lot 1 WB 1:500
mNeonGreen mouse Chromotek 32f6-100 Clone 32F6 Lot 90425021AB-01 IF 1:500
GFP mouse Roche 11814460001 Clones 7.1 and 13.1 Lot 47859600 WB 1:500
beta-actin mouse Sigma-Aldrich A5316 Clone AC-74 Lot 096M4855V WB 1:20 000
LC3B rabbit MBL PM036 Lot034 IF 1:500
LC3B rabbit Cell Signaling Technology 2775s Lot12, Lot13 WB 1:1000, IF 1:100
WIPI2 mouse abcam ab105459 Clone 2A2 Lot GR3225460-2 IF 1:200
p62 rabbit MBL PM045 Lot022 WB 1:10 000, IF 1:500
p62 guinea pig Progen GP62-C Lot 703241-03 IF 1:250
Ubiquitin mouse EMD Millipore Corp. 04-263 Clone FK2 Lot 3299923 IF 1:400
Ubiquitin-K63 rabbit Millipore 05-1308 Clone Apu3 Lot 2012973 IF 1:200
Tom20 mouse BD 612278 Clone 612278 Lot 8004895 IF 1:100
Atg13 rabbit Cell Signaling Technology #13468 Clone E1Y9V WB 1:1000

Alexa488-anti-mouse donkey Jackson 715-545-151 Lot 148532 1:500
Alexa488-anti-rabbit donkey Jackson 711-545-152 Lot 146871 1:500
Alexa568-anti-mouse donkey Molecular Probes A10037 Lot 2420698 1:500
Alexa568-anti-rabbit donkey Molecular Probes A10042 Lot 1891789 1:500
Alexa647-anti-mouse donkey Jackson 715-605-150 Lot 151088 1:500
Alexa647-anti-rabbit donkey Jackson 711- 605 -152 Lot 145376 1:500
Alexa647-anti-guinea pig donkey Jackson 706-605-148 Lot 101663 1:500
anti- rabbit IgG HRP goat Bio-Rad 1706515 Lot 64371828 WB 1:2000
Anti-rabbit HRP goat Jackson 111-035-144 Lot 157511 WB 1:5000
Anti-mouse HRP goat Jackson 115-035-003 Lot 158821 WB 1:5000
anti-rabbit IRDye 680RD donkey LI-COR Biosciences 926-68073 WB 1:5000
anti-mouse IRDye 680RD donkey LI-COR Biosciences 926-68072 WB 1:5000
anti-goat
IRDye 800CW donkey LI-COR Biosciences 926-32214 WB 1:5000
anti-rabbit IRDye 800CW donkey LI-COR Biosciences 926-32213 WB 1:5000
Anti-mouse IRDye 800CW donkey LI-COR Biosciences 926-32212 WB 1:5000

Validation

Antibodies were validated using either siRNA depletion, knock-out or functional tests

Eukaryotic cell lines

Policy information about cell lines and Sex and Gender in Research

Cell line source(s)

U2OS cells (ATCC: HTB-96), female origin
A431 (ATCC, Cat# CRL-1555, RRID:CVCL_0037), female origin
HeLa cells (obtained from Institute Curie, Paris, France), female origin
hTERT-RPE-1 cells (ATCC: CRL-4000), female origin
Sf9 (Novagen)

Authentication

Cells were authenticated by the Genotyping core facility at Oslo University hospital using Powerplex16 assays

Mycoplasma contamination

Original cell stocks were verified to be mycoplasma-free and were regularly tested, after manipulation (e.g. generation of clones or cell lines), cells were again verified to be mycoplasma-free.

Commonly misidentified lines
(See ICLAC register)

none

Flow Cytometry

Plots

Confirm that:

☒ The axis labels state the marker and fluorochrome used (e.g. CD4-FITC).

☒ The axis scales are clearly visible. Include numbers along axes only for bottom left plot of group (a 'group' is an analysis of identical markers).

☒ All plots are contour plots with outliers or pseudocolor plots.

☒ A numerical value for number of cells or percentage (with statistics) is provided.

Methodology

Sample preparation	U2OS and RPE-1 cells were detached by trypsin/EDTA and resuspended in complete cell culture medium for analysis on a BD LSR II Flow Cytometer.
Instrument	BD LSR II Flow Cytometer (BD Biosciences, San Jose, CA, USA) connected to the BD FACSDiva™ software (BD Biosciences, San Jose, CA, USA)
Software	FACS DIVA software was used for data collection
Cell population abundance	No sorting was performed, only gating for measurements; no specific cell populations were selected
Gating strategy	Gating strategy was performed as described in detail in Engedal N et al, 2022 (doi.org/10.1007/978-1-0716-2071-7_7). Briefly, live cells are identified, and dead cells and cell debris are excluded. Secondly, these gated cells are plotted for SSC-W versus SSC-A to set a gate around single cells. Thirdly, cells are plotted for the fluorescent signals obtained from excitation with the 407 nm laser versus the 561 nm laser. Finally, a gate is set around the mKeima positive population. A no-doxycycline control serves to identify the mKeima negative population.

☐ Tick this box to confirm that a figure exemplifying the gating strategy is provided in the Supplementary Information.
