# Supplementary material for: ATPase activity of DFCP1 controls selective autophagy
Source: Nat Commun. 2023 Jul 8;14:4051. doi: 10.1038/s41467-023-39641-9 (PMC10329651; doi:10.1038/s41467-023-39641-9)
Supplement: Supplementary file 11 — Supplementary Data [file 41467_2023_39641_MOESM11_ESM.zip › StructuralPredictions/Phyre_output/DFCP1_PHYRE__report.pdf]

Phyre2

Email	k.schink@gmail.com
Description	DFCP1_2__
Date	Thu Jan 21 21:38:25 GMT 2021
Unique Job ID	3b8639168e111538

Detailed template information

#	Template	Alignment Coverage	3D Model	Confidence	% i.d.	Template Information
1	c1f5nA_	Alignment	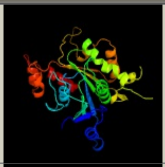	100.0	17	**PDB header:**signaling protein **Chain:** A: **PDB Molecule:**interferon-induced guanylate-binding protein 1; **PDBTitle:** human guanylate binding protein-1 in complex with the gtp analogue,2 gmppnp.
2	c3q5dA_	Alignment	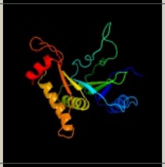	100.0	17	**PDB header:**hydrolase **Chain:** A: **PDB Molecule:**atlastin-1; **PDBTitle:** crystal structure of human atlastin-1 (residues 1-447) bound to gdp,2 crystal form 1
3	c5vgrA_	Alignment	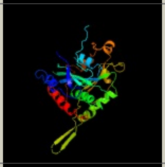	100.0	15	**PDB header:**hydrolase **Chain:** A: **PDB Molecule:**atlastin-3; **PDBTitle:** human atlastin-3, gdp-bound
4	c5ca8A_	Alignment	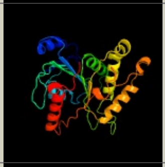	100.0	19	**PDB header:**hydrolase **Chain:** A: **PDB Molecule:**protein sey1; **PDBTitle:** structures of the yeast dynamin-like gtpase sey1p in complex with gdp
5	c3x1dA_	Alignment	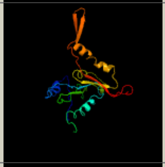	100.0	18	**PDB header:**hydrolase **Chain:** A: **PDB Molecule:**atlastin; **PDBTitle:** crystal structure of atlastin from drosophila melanogaster
6	c2b92A_	Alignment	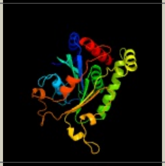	100.0	19	**PDB header:**signaling protein **Chain:** A: **PDB Molecule:**interferon-induced guanylate-binding protein 1; **PDBTitle:** crystal-structure of the n-terminal large gtpase domain of human2 guanylate binding protein 1 (hgbp1) in complex with gdp/alf3
7	c3q5eG_	Alignment	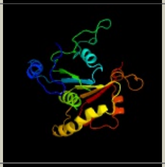	100.0	16	**PDB header:**hydrolase **Chain:** G: **PDB Molecule:**atlastin-1; **PDBTitle:** crystal structure of human atlastin-1 (residues 1-447) bound to gdp,2 crystal form 2
8	d1f5na2	Alignment	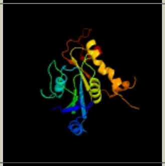	100.0	21	**Fold:**P-loop containing nucleoside triphosphate hydrolases **Superfamily:**P-loop containing nucleoside triphosphate hydrolases **Family:**G proteins
9	c6sytC_	Alignment	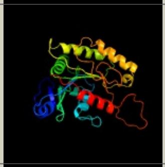	100.0	15	**PDB header:**signaling protein **Chain:** C: **PDB Molecule:**protein smg9; **PDBTitle:** structure of the smg1-smg8-smg9 complex
10	c5nkkB_	Alignment	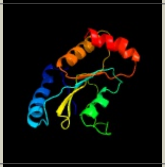	99.8	19	**PDB header:**transcription **Chain:** B: **PDB Molecule:**protein smg-9; **PDBTitle:** smg8-smg9 complex gdp bound
11	c4aurA_	Alignment	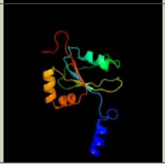	99.3	15	**PDB header:**hydrolase **Chain:** A: **PDB Molecule:**leoa; **PDBTitle:** leoa bacterial dynamin gtpase from etec

12	c3zjcC_	Alignment	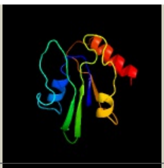	99.2	19	**PDB header:**hydrolase<br>**Chain:** C: **PDB Molecule:**gtpase imap family member 7;<br>**PDBTitle:** crystal structure of gmppnp-bound human gimap7 l100q variant
13	c2xtnA_	Alignment	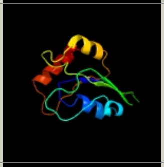	99.1	15	**PDB header:**immune system<br>**Chain:** A: **PDB Molecule:**gtpase imap family member 2;<br>**PDBTitle:** crystal structure of gtp-bound human gimap2, amino acid2 residues 1-234
14	c5oxfB_	Alignment	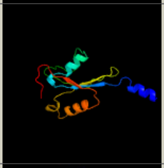	99.0	24	**PDB header:**lipid binding protein<br>**Chain:** B: **PDB Molecule:**gtp-binding protein;<br>**PDBTitle:** an oligomerised bacterial dynamin pair provides a mechanism for the2 long range sensing and tethering of membranes
15	c2xtpA_	Alignment	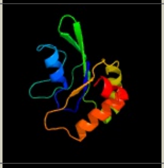	99.0	14	**PDB header:**immune system<br>**Chain:** A: **PDB Molecule:**gtpase imap family member 2;<br>**PDBTitle:** crystal structure of nucleotide-free human gimap2, amino2 acid residues 1-260
16	c5owvA_	Alignment	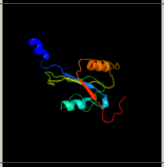	98.9	34	**PDB header:**lipid binding protein<br>**Chain:** A: **PDB Molecule:**gtp-binding protein;<br>**PDBTitle:** an oligomerised bacterial dynamin pair provides a mechanism for the2 long-range sensing and tethering of membranes
17	c2j3eA_	Alignment	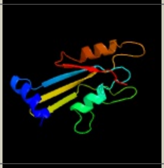	98.9	23	**PDB header:**protein transport<br>**Chain:** A: **PDB Molecule:**t7i23.11 protein;<br>**PDBTitle:** dimerization is important for the gtpase activity of2 chloroplast translocon components attoc33 and pstoc159
18	c5mvfA_	Alignment	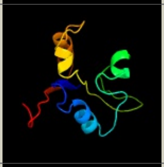	98.9	18	**PDB header:**endocytosis<br>**Chain:** A: **PDB Molecule:**eh domain-containing protein 4;<br>**PDBTitle:** active structure of ehd4 complexed with adp
19	d1h65a_	Alignment	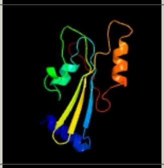	98.9	23	**Fold:**P-loop containing nucleoside triphosphate hydrolases<br>**Superfamily:**P-loop containing nucleoside triphosphate hydrolases<br>**Family:**G proteins
20	c2j69D_	Alignment	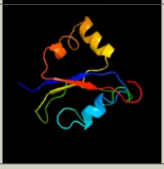	98.8	29	**PDB header:**hydrolase<br>**Chain:** D: **PDB Molecule:**bacterial dynamin-like protein;<br>**PDBTitle:** bacterial dynamin-like protein bdlp
21	c3k53B_	Alignment	not modelled	98.8	19	**PDB header:**metal transport<br>**Chain:** B: **PDB Molecule:**ferrous iron transport protein b;<br>**PDBTitle:** crystal structure of nfeob from p. furiosus
22	c2qptA_	Alignment	not modelled	98.8	18	**PDB header:**endocytosis<br>**Chain:** A: **PDB Molecule:**eh domain-containing protein-2;<br>**PDBTitle:** crystal structure of an ehd atpase involved in membrane remodelling
23	d1tq4a_	Alignment	not modelled	98.7	18	**Fold:**P-loop containing nucleoside triphosphate hydrolases<br>**Superfamily:**P-loop containing nucleoside triphosphate hydrolases<br>**Family:**G proteins
24	c3i8sC_	Alignment	not modelled	98.7	20	**PDB header:**transport protein<br>**Chain:** C: **PDB Molecule:**ferrous iron transport protein b;<br>**PDBTitle:** structure of the cytosolic domain of e. coli feob, nucleotide-free2 form
25	c3a1vB_	Alignment	not modelled	98.7	10	**PDB header:**transport protein<br>**Chain:** B: **PDB Molecule:**iron(ii) transport protein b;<br>**PDBTitle:** crystal structue of the cytosolic domain of t. maritima feob2 iron iransporter in apo form
26	c3ievA_	Alignment	not modelled	98.7	17	**PDB header:**nucleotide binding protein/rna<br>**Chain:** A: **PDB Molecule:**gtp-binding protein era;<br>**PDBTitle:** crystal structure of era in complex with mggnp and the 3' end of 16s2 rrna
27	c3ibyA_	Alignment	not modelled	98.7	16	**PDB header:**transport protein<br>**Chain:** A: **PDB Molecule:**ferrous iron transport protein b;<br>**PDBTitle:** structure of cytosolic domain of l. pneumophila feob
28	c3lx8A_	Alignment	not modelled	98.7	12	**PDB header:**metal transport<br>**Chain:** A: **PDB Molecule:**ferrous iron uptake transporter protein b;<br>**PDBTitle:** crystal structure of gdp-bound nfeob from s. thermophilus
						**PDB header:**lipid binding protein

29	c5owvC_	Alignment	not modelled	98.6	22	**Chain:** C: **PDB Molecule:**gtp-binding protein;<br>**PDBTitle:** an oligomerised bacterial dynamin pair provides a mechanism for the2 long-range sensing and tethering of membranes
30	c5gnuA_	Alignment	not modelled	98.6	25	**PDB header:**hydrolase<br>**Chain:** A: **PDB Molecule:**mitofusin-1;<br>**PDBTitle:** the structure of mini-mfn1 apo
31	c3lxwA_	Alignment	not modelled	98.6	21	**PDB header:**immune system<br>**Chain:** A: **PDB Molecule:**gtpase imap family member 1;<br>**PDBTitle:** crystal structure of human gtpase imap family member 1
32	c6jflB_	Alignment	not modelled	98.6	22	**PDB header:**membrane protein<br>**Chain:** B: **PDB Molecule:**mitofusin-2,cdna flj57997, highly similar to transmembrane<br>**PDBTitle:** nucleotide-free mitofusin2 (mfn2)
33	c5ady6_	Alignment	not modelled	98.5	17	**PDB header:**ribosome<br>**Chain:** 6: **PDB Molecule:**gtpase hflx;<br>**PDBTitle:** cryo-em structures of the 50s ribosome subunit bound with hflx
34	c6j73B_	Alignment	not modelled	98.5	22	**PDB header:**hydrolase<br>**Chain:** B: **PDB Molecule:**isoniazid inducible gene protein inia;<br>**PDBTitle:** crystal structure of inia from mycobacterium smegmatis
35	d1ni3a1	Alignment	not modelled	98.5	17	**Fold:**P-loop containing nucleoside triphosphate hydrolases<br>**Superfamily:**P-loop containing nucleoside triphosphate hydrolases<br>**Family:**G proteins
36	c1wf3A_	Alignment	not modelled	98.5	18	**PDB header:**hydrolase<br>**Chain:** A: **PDB Molecule:**gtp-binding protein;<br>**PDBTitle:** crystal structure of gtp-binding protein tt1341 from thermus2 thermophilus hb8
37	c3p1jC_	Alignment	not modelled	98.5	16	**PDB header:**hydrolase<br>**Chain:** C: **PDB Molecule:**gtpase imap family member 2;<br>**PDBTitle:** crystal structure of human gtpase imap family member 2 in the2 nucleotide-free state
38	c6djqA_	Alignment	not modelled	98.4	17	**PDB header:**hydrolase<br>**Chain:** A: **PDB Molecule:**vps1 gtpase-bse;<br>**PDBTitle:** vps1 gtpase-bse fusion complexed with gdp.alf4-
39	c1mkyA_	Alignment	not modelled	98.4	20	**PDB header:**ligand binding protein<br>**Chain:** A: **PDB Molecule:**probable gtp-binding protein enga;<br>**PDBTitle:** structural analysis of the domain interactions in der, a switch2 protein containing two gtpase domains
40	c2qthA_	Alignment	not modelled	98.4	17	**PDB header:**nucleotide binding protein<br>**Chain:** A: **PDB Molecule:**gtp-binding protein;<br>**PDBTitle:** crystal structure of a gtp-binding protein from the hyperthermophilic2 archaeon sulfolobus solfataricus in complex with gdp
41	c3w5iB_	Alignment	not modelled	98.4	14	**PDB header:**metal transport<br>**Chain:** B: **PDB Molecule:**ferrous iron transport protein b;<br>**PDBTitle:** crystal structure of nfeob from gallionella capsiferriformans
42	c5nkmE_	Alignment	not modelled	98.4	18	**PDB header:**rna binding protein<br>**Chain:** E: **PDB Molecule:**protein smg-8;<br>**PDBTitle:** smg8-smg9 complex
43	c6jtgA_	Alignment	not modelled	98.4	14	**PDB header:**hydrolase<br>**Chain:** A: **PDB Molecule:**dynamin-like 120 kda protein, mitochondrial,opa1 protein;<br>**PDBTitle:** structural insights into g domain dimerization and pathogenic2 mutations of opa1
44	c2e87A_	Alignment	not modelled	98.4	21	**PDB header:**structural genomics, unknown function<br>**Chain:** A: **PDB Molecule:**hypothetical protein ph1320;<br>**PDBTitle:** crystal structure of hypothetical gtp-binding protein ph1320 from2 pyrococcus horikoshii ot3, in complex with gdp
45	d2akab1	Alignment	not modelled	98.4	19	**Fold:**P-loop containing nucleoside triphosphate hydrolases<br>**Superfamily:**P-loop containing nucleoside triphosphate hydrolases<br>**Family:**G proteins
46	c1xzqA_	Alignment	not modelled	98.4	17	**PDB header:**hydrolase<br>**Chain:** A: **PDB Molecule:**probable trna modification gtpase trme;<br>**PDBTitle:** structure of the gtp-binding protein trme from thermotoga2 maritima complexed with 5-formyl-thf
47	d1jala1	Alignment	not modelled	98.4	18	**Fold:**P-loop containing nucleoside triphosphate hydrolases<br>**Superfamily:**P-loop containing nucleoside triphosphate hydrolases<br>**Family:**G proteins
48	c1udxA_	Alignment	not modelled	98.3	18	**PDB header:**protein binding<br>**Chain:** A: **PDB Molecule:**the gtp-binding protein obg;<br>**PDBTitle:** crystal structure of the conserved protein tt1381 from thermus2 thermophilus hb8
49	d1jwyb_	Alignment	not modelled	98.3	16	**Fold:**P-loop containing nucleoside triphosphate hydrolases<br>**Superfamily:**P-loop containing nucleoside triphosphate hydrolases<br>**Family:**G proteins
50	c5dn8A_	Alignment	not modelled	98.3	16	**PDB header:**gtp-binding protein<br>**Chain:** A: **PDB Molecule:**gtpase der;<br>**PDBTitle:** 1.76 angstrom crystal structure of gtp-binding protein der from2 coxiella burnetii in complex with gdp.
51	c1egaB_	Alignment	not modelled	98.3	20	**PDB header:**hydrolase<br>**Chain:** B: **PDB Molecule:**protein (gtp-binding protein era);<br>**PDBTitle:** crystal structure of a widely conserved gtpase era
52	c6em5b_	Alignment	not modelled	98.2	21	**PDB header:**ribosome<br>**Chain:** B: **PDB Molecule:**60s ribosomal protein l3;<br>**PDBTitle:** state d architectural model (nsa1-tap flag-ytm1) - visualizing the2 assembly pathway of nucleolar pre-60s ribosomes
53	c3t5dC_	Alignment	not modelled	98.2	22	**PDB header:**signaling protein<br>**Chain:** C: **PDB Molecule:**septin-7;<br>**PDBTitle:** crystal structure of septin 7 in complex with gdp
54	c6sytB_	Alignment	not modelled	98.2	17	**PDB header:**signaling protein<br>**Chain:** B: **PDB Molecule:**protein smg8;<br>**PDBTitle:** structure of the smg1-smg8-smg9 complex

55	c3sopB_	Alignment	not modelled	98.2	28	**PDB header:**hydrolase<br>**Chain:** B: **PDB Molecule:**neuronal-specific septin-3;<br>**PDBTitle:** crystal structure of human septin 3 gtpase domain
56	c4yqfA_	Alignment	not modelled	98.2	24	**PDB header:**hydrolase<br>**Chain:** A: **PDB Molecule:**septin-9;<br>**PDBTitle:** gtpase domain of human septin 9
57	c4bejB_	Alignment	not modelled	98.2	20	**PDB header:**hydrolase<br>**Chain:** B: **PDB Molecule:**dynamin 1-like protein;<br>**PDBTitle:** nucleotide-free dynamin 1-like protein (dnm1l, drp1, dlp1)
58	c2qagB_	Alignment	not modelled	98.2	21	**PDB header:**cell cycle, structural protein<br>**Chain:** B: **PDB Molecule:**septin-6;<br>**PDBTitle:** crystal structure of human septin trimer 2/6/7
59	c6xrsB_	Alignment	not modelled	98.2	17	**PDB header:**hydrolase<br>**Chain:** B: **PDB Molecule:**gtpase der;<br>**PDBTitle:** crystal structure of a gtp-binding protein enga (der homolog) from2 neisseria gonorrhoeae bound to gdp
60	c6mqbA_	Alignment	not modelled	98.2	22	**PDB header:**structural protein<br>**Chain:** A: **PDB Molecule:**septin-12;<br>**PDBTitle:** crystal structure of gtpase domain of human septin 12 in complex with2 gmppnp in space group c2221
61	c4h1vA_	Alignment	not modelled	98.1	16	**PDB header:**hydrolase<br>**Chain:** A: **PDB Molecule:**dynamin-1-like protein;<br>**PDBTitle:** gmp-pnp bound dynamin-1-like protein gtpase-ged fusion
62	c4dheA_	Alignment	not modelled	98.1	15	**PDB header:**cell cycle<br>**Chain:** A: **PDB Molecule:**probable gtp-binding protein engb;<br>**PDBTitle:** crystal structure of a probable gtp-binding protein engb from2 burkholderia thailandensis
63	c4a9aB_	Alignment	not modelled	98.1	19	**PDB header:**translation<br>**Chain:** B: **PDB Molecule:**ribosome-interacting gtpase 1;<br>**PDBTitle:** structure of rbg1 in complex with tma46 dfrp domain
64	c4whjA_	Alignment	not modelled	98.1	19	**PDB header:**antiviral protein, hydrolase<br>**Chain:** A: **PDB Molecule:**interferon-induced gtp-binding protein mx2;<br>**PDBTitle:** myxovirus resistance protein 2 (mxb)
65	c2x2fD_	Alignment	not modelled	98.0	20	**PDB header:**hydrolase<br>**Chain:** D: **PDB Molecule:**dynamin-1;<br>**PDBTitle:** dynamin 1 gtpase dimer, short axis form
66	d2bv3a2	Alignment	not modelled	98.0	17	**Fold:**P-loop containing nucleoside triphosphate hydrolases<br>**Superfamily:**P-loop containing nucleoside triphosphate hydrolases<br>**Family:**G proteins
67	d2dy1a2	Alignment	not modelled	98.0	19	**Fold:**P-loop containing nucleoside triphosphate hydrolases<br>**Superfamily:**P-loop containing nucleoside triphosphate hydrolases<br>**Family:**G proteins
68	c2qagC_	Alignment	not modelled	98.0	23	**PDB header:**cell cycle, structural protein<br>**Chain:** C: **PDB Molecule:**septin-7;<br>**PDBTitle:** crystal structure of human septin trimer 2/6/7
69	c3lxxA_	Alignment	not modelled	98.0	17	**PDB header:**immune system<br>**Chain:** A: **PDB Molecule:**gtpase imap family member 4;<br>**PDBTitle:** crystal structure of human gtpase imap family member 4
70	c1ni3A_	Alignment	not modelled	98.0	16	**PDB header:**hydrolase<br>**Chain:** A: **PDB Molecule:**ychf gtp-binding protein;<br>**PDBTitle:** structure of the schizosaccharomyces pombe ychf gtpase
71	c4csu9_	Alignment	not modelled	98.0	12	**PDB header:**ribosome<br>**Chain:** 9: **PDB Molecule:**gtpase obge/cgta;<br>**PDBTitle:** cryo-em structures of the 50s ribosome subunit bound with obge
72	c4lpsA_	Alignment	not modelled	98.0	13	**PDB header:**metal binding protein<br>**Chain:** A: **PDB Molecule:**hydrogenase/urease nickel incorporation protein hypb;<br>**PDBTitle:** crystal structure of hypb from helicobacter pylori in complex with2 nickel
73	c4p4sB_	Alignment	not modelled	97.9	19	**PDB header:**antiviral protein/hydrolase<br>**Chain:** B: **PDB Molecule:**interferon-induced gtp-binding protein mx1;<br>**PDBTitle:** gmppcp-bound stalkless-mxa
74	d1lnza2	Alignment	not modelled	97.9	14	**Fold:**P-loop containing nucleoside triphosphate hydrolases<br>**Superfamily:**P-loop containing nucleoside triphosphate hydrolases<br>**Family:**G proteins
75	c1u0lB_	Alignment	not modelled	97.9	17	**PDB header:**hydrolase<br>**Chain:** B: **PDB Molecule:**probable gtpase engc;<br>**PDBTitle:** crystal structure of yjeq from thermotoga maritima
76	d1wb1a4	Alignment	not modelled	97.9	17	**Fold:**P-loop containing nucleoside triphosphate hydrolases<br>**Superfamily:**P-loop containing nucleoside triphosphate hydrolases<br>**Family:**G proteins
77	d1puja_	Alignment	not modelled	97.9	15	**Fold:**P-loop containing nucleoside triphosphate hydrolases<br>**Superfamily:**P-loop containing nucleoside triphosphate hydrolases<br>**Family:**G proteins
78	c5ee1A_	Alignment	not modelled	97.9	15	**PDB header:**hydrolase<br>**Chain:** A: **PDB Molecule:**obg-like atpase 1;<br>**PDBTitle:** crystal structure of osychf1 at ph 7.85
79	c2hf9A_	Alignment	not modelled	97.8	13	**PDB header:**hydrolase, metal binding protein<br>**Chain:** A: **PDB Molecule:**probable hydrogenase nickel incorporation<br>**PDBTitle:** crystal structure of hypb from methanocaldococcus2 jannaschii in the triphosphate form
80	c2wjjB_	Alignment	not modelled	97.8	13	**PDB header:**metal transport<br>**Chain:** B: **PDB Molecule:**ferrous iron transport protein b homolog;<br>**PDBTitle:** structure and function of the feob g-domain from2 methanococcus jannaschii
81	c3gehA_	Alignment	not modelled	97.8	25	**PDB header:**hydrolase<br>**Chain:** A: **PDB Molecule:**trna modification gtpase mnme;<br>**PDBTitle:** crystal structure of mnme from nostoc in complex with

						gdp, folinic2 acid and zn
82	d1u0la2	Alignment	not modelled	97.8	16	**Fold:**P-loop containing nucleoside triphosphate hydrolases **Superfamily:**P-loop containing nucleoside triphosphate hydrolases **Family:**G proteins
83	c2rcnA_	Alignment	not modelled	97.8	17	**PDB header:**hydrolase **Chain:** A: **PDB Molecule:**probable gtpase engc; **PDBTitle:** crystal structure of the ribosomal interacting gtpase yjeq from the2 enterobacterial species salmonella typhimurium.
84	c4a2iV_	Alignment	not modelled	97.8	19	**PDB header:**ribosome/hydrolase **Chain:** V: **PDB Molecule:**putative ribosome biogenesis gtpase rsga; **PDBTitle:** cryo-electron microscopy structure of the 30s subunit in complex with2 the yjeq biogenesis factor
85	c3j65o_	Alignment	not modelled	97.8	23	**PDB header:**ribosome **Chain:** O: **PDB Molecule:**60s ribosomal protein l15; **PDBTitle:** arx1 pre-60s particle. this entry contains the r-proteins and2 biogenesis factors.
86	c1jalA_	Alignment	not modelled	97.7	19	**PDB header:**structural genomics, unknown function **Chain:** A: **PDB Molecule:**ychf protein; **PDBTitle:** ychf protein (hi0393)
87	c3t35A_	Alignment	not modelled	97.7	14	**PDB header:**motor protein **Chain:** A: **PDB Molecule:**dynamin-related protein 1a, linker, dynamin-related protein **PDBTitle:** arabidopsis thaliana dynamin-related protein 1a in postfission state
88	c5irrB_	Alignment	not modelled	97.7	12	**PDB header:**hydrolase **Chain:** B: **PDB Molecule:**septin-like protein; **PDBTitle:** crystal structure of septin gtpase domain from chlamydomonas2 reinhardtii
89	c2yv5A_	Alignment	not modelled	97.7	18	**PDB header:**hydrolase **Chain:** A: **PDB Molecule:**yjeq protein; **PDBTitle:** crystal structure of yjeq from aquifex aeolicus
90	c6vs4B_	Alignment	not modelled	97.7	21	**PDB header:**hydrolase **Chain:** B: **PDB Molecule:**small copii coat gtpase sar1; **PDBTitle:** crystal structure of adp ribosylation factor-like gtp binding protein2 /small copii coat gtpase sar1 from encephalitozoon cuniculi in3 complex with gdp
91	c5a3fD_	Alignment	not modelled	97.7	21	**PDB header:**endocytosis **Chain:** D: **PDB Molecule:**dynamin 3; **PDBTitle:** crystal structure of the dynamin tetramer
92	c3ftqA_	Alignment	not modelled	97.7	21	**PDB header:**cell cycle **Chain:** A: **PDB Molecule:**septin-2; **PDBTitle:** crystal structure of septin 2 in complex with gppnhp and2 mg2+
93	c2q3fB_	Alignment	not modelled	97.7	10	**PDB header:**protein binding **Chain:** B: **PDB Molecule:**ras-related gtp-binding protein d; **PDBTitle:** x-ray crystal structure of putative human ras-related gtp2 binding d in complex with gmppnp
94	c2ohfA_	Alignment	not modelled	97.7	17	**PDB header:**hydrolase **Chain:** A: **PDB Molecule:**gtp-binding protein 9; **PDBTitle:** crystal structure of human ola1 in complex with amppcp
95	c3j8gX_	Alignment	not modelled	97.7	17	**PDB header:**ribosome **Chain:** X: **PDB Molecule:**gtpase der; **PDBTitle:** electron cryo-microscopy structure of enga bound with the 50s2 ribosomal subunit
96	c2lkcA_	Alignment	not modelled	97.7	20	**PDB header:**translation **Chain:** A: **PDB Molecule:**translation initiation factor if-2; **PDBTitle:** free b.st if2-g2
97	c3qq5A_	Alignment	not modelled	97.6	21	**PDB header:**oxidoreductase **Chain:** A: **PDB Molecule:**small gtp-binding protein; **PDBTitle:** crystal structure of the [fefe]-hydrogenase maturation protein hydf
98	d1egaa1	Alignment	not modelled	97.6	19	**Fold:**P-loop containing nucleoside triphosphate hydrolases **Superfamily:**P-loop containing nucleoside triphosphate hydrolases **Family:**G proteins
99	c3nxsA_	Alignment	not modelled	97.6	18	**PDB header:**transport protein **Chain:** A: **PDB Molecule:**lao/ao transport system atpase; **PDBTitle:** crystal structure of lao/ao transport system from mycobacterium2 smegmatis bound to gdp
100	c4p4sA_	Alignment	not modelled	97.6	19	**PDB header:**antiviral protein/hydrolase **Chain:** A: **PDB Molecule:**interferon-induced gtp-binding protein mx1; **PDBTitle:** gmppcp-bound stalkless-mxa
101	d1wf3a1	Alignment	not modelled	97.6	14	**Fold:**P-loop containing nucleoside triphosphate hydrolases **Superfamily:**P-loop containing nucleoside triphosphate hydrolases **Family:**G proteins
102	c3pqcA_	Alignment	not modelled	97.6	23	**PDB header:**hydrolase **Chain:** A: **PDB Molecule:**probable gtp-binding protein engb; **PDBTitle:** crystal structure of thermotoga maritima ribosome biogenesis gtp-2 binding protein engb (ysxc/yiha) in complex with gdp
103	c2qa5A_	Alignment	not modelled	97.6	26	**PDB header:**cell cycle, structural protein **Chain:** A: **PDB Molecule:**septin-2; **PDBTitle:** crystal structure of sept2 g-domain
104	c6g15A_	Alignment	not modelled	97.6	21	**PDB header:**rna binding protein **Chain:** A: **PDB Molecule:**ribosome biogenesis gtpase a; **PDBTitle:** crystal structure of pppgpp bound rbga from s. aureus
105	d2gj8a1	Alignment	not modelled	97.6	20	**Fold:**P-loop containing nucleoside triphosphate hydrolases **Superfamily:**P-loop containing nucleoside triphosphate hydrolases **Family:**G proteins
106	c2dwqB_	Alignment	not modelled	97.5	18	**PDB header:**hydrolase **Chain:** B: **PDB Molecule:**gtp-binding protein; **PDBTitle:** thermus thermophilus ychf gtp-binding protein
107	d1zunb3	Alignment	not modelled	97.5	17	**Fold:**P-loop containing nucleoside triphosphate hydrolases **Superfamily:**P-loop containing nucleoside triphosphate hydrolases

						**Family:**G proteins
108	c2hjgA_	Alignment	not modelled	97.5	18	**PDB header:**hydrolase<br>**Chain:** A: **PDB Molecule:**gtp-binding protein enga;<br>**PDBTitle:** the crystal structure of the b. subtilis yphc gtpase in complex with2 gdp
109	d2qm8a1	Alignment	not modelled	97.5	19	**Fold:**P-loop containing nucleoside triphosphate hydrolases<br>**Superfamily:**P-loop containing nucleoside triphosphate hydrolases<br>**Family:**Nitrogenase iron protein-like
110	d2p67a1	Alignment	not modelled	97.5	15	**Fold:**P-loop containing nucleoside triphosphate hydrolases<br>**Superfamily:**P-loop containing nucleoside triphosphate hydrolases<br>**Family:**Nitrogenase iron protein-like
111	c3r7wC_	Alignment	not modelled	97.5	10	**PDB header:**protein transport<br>**Chain:** C: **PDB Molecule:**gtp-binding protein gtr1;<br>**PDBTitle:** crystal structure of gtr1p-gtr2p complex
112	c2wwwB_	Alignment	not modelled	97.5	15	**PDB header:**transport protein<br>**Chain:** B: **PDB Molecule:**methylmalonic aciduria type a protein,<br>**PDBTitle:** crystal structure of methylmalonic acidemia type a protein
113	c3cnlA_	Alignment	not modelled	97.5	25	**PDB header:**signaling protein<br>**Chain:** A: **PDB Molecule:**putative uncharacterized protein;<br>**PDBTitle:** crystal structure of gnp-bound ylqf from t. maritima
114	c3md0A_	Alignment	not modelled	97.5	16	**PDB header:**transport protein<br>**Chain:** A: **PDB Molecule:**arginine/ornithine transport system atpase;<br>**PDBTitle:** crystal structure of arginine/ornithine transport system atpase from2 mycobacterium tuberculosis bound to gdp (a ras-like gtpase3 superfamily protein)
115	c3zvrA_	Alignment	not modelled	97.4	21	**PDB header:**hydrolase<br>**Chain:** A: **PDB Molecule:**dynamin-1;<br>**PDBTitle:** crystal structure of dynamin
116	c1lnzA_	Alignment	not modelled	97.4	13	**PDB header:**cell cycle<br>**Chain:** A: **PDB Molecule:**spo0b-associated gtp-binding protein;<br>**PDBTitle:** structure of the obg gtp-binding protein
117	c6h4dA_	Alignment	not modelled	97.4	17	**PDB header:**rna binding protein<br>**Chain:** A: **PDB Molecule:**small ribosomal subunit biogenesis gtpase rsga;<br>**PDBTitle:** crystal structure of rsga from pseudomonas aeruginosa
118	d1udxa2	Alignment	not modelled	97.4	15	**Fold:**P-loop containing nucleoside triphosphate hydrolases<br>**Superfamily:**P-loop containing nucleoside triphosphate hydrolases<br>**Family:**G proteins
119	c6ql4B_	Alignment	not modelled	97.4	19	**PDB header:**motor protein<br>**Chain:** B: **PDB Molecule:**putative mitochondrial dynamin protein;<br>**PDBTitle:** crystal structure of nucleotide-free mgm1
120	c3a1wA_	Alignment	not modelled	97.4	11	**PDB header:**transport protein<br>**Chain:** A: **PDB Molecule:**iron(ii) transport protein b;<br>**PDBTitle:** crystal structue of the g domain of t. maritima feob iron2 iransporter
