# Supplementary figures and images for: ATPase activity of DFCP1 controls selective autophagy
Source: Nat Commun. 2023 Jul 8;14:4051. doi: 10.1038/s41467-023-39641-9 (PMC10329651; doi:10.1038/s41467-023-39641-9)

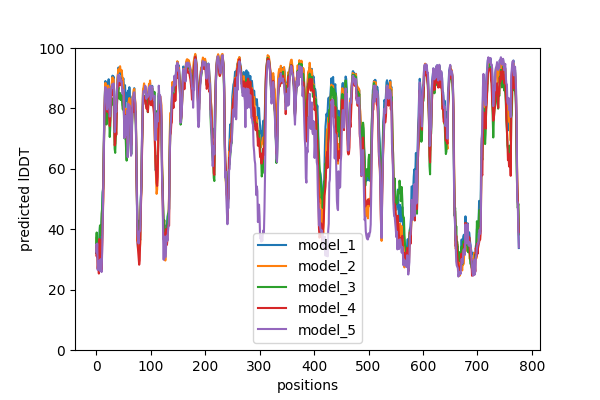

Supplement: Supplementary file 11 — Supplementary Data [file 41467_2023_39641_MOESM11_ESM.zip › StructuralPredictions/Alphafold_output/ZF1_HUMAN_lDDT.png]

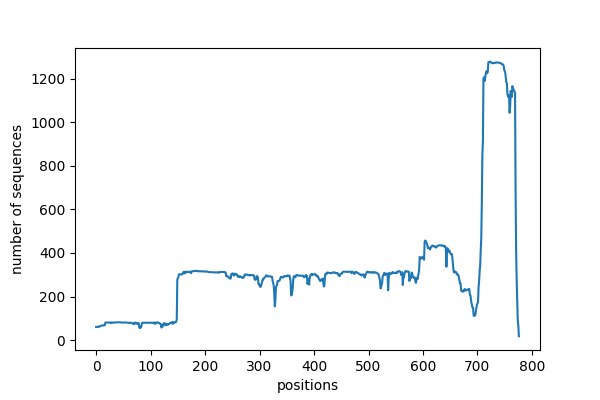

Supplement: Supplementary file 11 — Supplementary Data [file 41467_2023_39641_MOESM11_ESM.zip › StructuralPredictions/Alphafold_output/ZF1_HUMAN_msa_coverage.png]
